# Identification of a Tumor Immunological Phenotype-Related Gene Signature for Predicting Prognosis, Immunotherapy Efficacy, and Drug Candidates in Hepatocellular Carcinoma

**DOI:** 10.3389/fimmu.2022.862527

**Published:** 2022-04-12

**Authors:** Yuqin Tang, Chengbin Guo, Zhao Yang, Yumei Wang, Yongqiang Zhang, Dong Wang

**Affiliations:** ^1^ State Key Laboratory of Southwestern Chinese Medicine Resources, School of Basic Medical Sciences, Chengdu University of Traditional Chinese Medicine, Chengdu, China; ^2^ Faculty of Medicine, Macau University of Science and Technology, Macau, China; ^3^ West China School of Medicine, West China Hospital, Sichuan University, Chengdu, China; ^4^ Guangzhou Women and Children’s Medical Center, Guangzhou Medical University, Guangzhou, China

**Keywords:** hepatocellular carcinoma, tumor immunological phenotype, immunotherapy efficacy, immune infiltration, prognosis, molecular docking

## Abstract

Hepatocellular carcinoma (HCC) is the predominant subtype of primary liver cancer and represents a highly heterogeneous disease, making it hard to predict the prognosis and therapy efficacy. Here, we established a novel tumor immunological phenotype-related gene index (TIPRGPI) consisting of 11 genes by Univariate Cox regression and the least absolute shrinkage and selection operator (LASSO) algorithm to predict HCC prognosis and immunotherapy response. TIPRGPI was validated in multiple datasets and exhibited outstanding performance in predicting the overall survival of HCC. Multivariate analysis verified it as an independent predictor and a TIPRGPI-integrated nomogram was constructed to provide a quantitative tool for clinical practice. Distinct mutation profiles, hallmark pathways, and infiltration of immune cells in tumor microenvironment were shown between the TIPRGPI high and low-risk groups. Notably, significant differences in tumor immunogenicity and tumor immune dysfunction and exclusion (TIDE) were observed between the two risk groups, suggesting a better response to immune checkpoint blockade (ICB) therapy of the low-risk group. Besides, six potential drugs binding to the core target of the TIPRGPI signature were predicted *via* molecular docking. Taken together, our study shows that the proposed TIPRGPI was a reliable signature to predict the risk classification, immunotherapy response, and drugs candidate with potential application in the clinical decision and treatment of HCC. The novel “TIP genes”-guided strategy for predicting the survival and immunotherapy efficacy, we reported here, might be also applied to more cancers other than HCC.

## Introduction

Liver cancer is one of the deadliest malignancies in the world and hepatocellular carcinoma (HCC) is the dominant type, accounting for ~75% of all cases (1). In the past decade, despite the great progress of surveillance, diagnosis and management in HCC, the mortality rate of HCC remains unacceptably high (2, 3). Due to the poor prognosis, the incidence and mortality rates of HCC are roughly equivalent (4). In 2018, the incidence rate per 100,000 in Eastern Asia was 17.7, whereas the corresponding mortality rate was 16.0 (5). The high prevalence and poor survival of HCC largely result from the heterogeneity of pathogenic factors, treatment responses, and molecular profiles. For instance, multiple factors, including chronic infections of Hepatitis B virus (HBV) or Hepatitis C virus (HCV), alcohol consumption, metabolic syndrome are strong causes for the incidence of HCC (2, 6, 7). While the HBV vaccine has been introduced by a number of countries to eliminate HBV-related HCC, there is still no vaccine available for HCV-related HCC and nonviral HCC (4). The presently used clinical characteristics including the tumor-node-metastasis (TNM) staging system, vascular invasion, and tumor burden status are limited in predicting the prognosis and treatment sensitivities for HCC (8, 9). Thus, novel prognostic classifiers or therapeutic biomarkers are urgently needed to improve the clinical benefits of HCC patients.

Tumor immune microenvironment (TIME) is proven to play a vital role in tumorigenesis and development (10). Immunotherapy with immune checkpoint inhibitors (ICIs), reversing the inactivation of immune cells to eliminate tumor cells, has emerged as a promising therapy for a variety of cancers in recent years (11). Multiple ICIs were approved for cancer therapy such as nivolumab, pembrolizumab, and cemiplimab targeting programmed death-1 (PD-1) and ipilimumab targeting cytotoxic T-lymphocyte-associated protein 4 (CTLA-4) (12). More agents targeting novel immune checkpoints such as T cell immunoglobulin and mucin-domain containing-3 (TIM-3), lymphocyte activation gene-3 (LAG-3), and T cell immunoglobulin and ITIM domain (TIGIT) are under investigation to expand the scope of immunotherapy (13–15). Adequate evidences suggested that chronic inflammation was a major risk factor for the development of HCC and immunotherapy might be the ideal approach to improve the prognosis of HCC (16, 17). The composition of tumor microenvironment (TME) of HCC is complex, in which a number of immune and stromal cells interact to form an immunosuppressive microenvironment and eventually lead to a worse prognosis of HCC (11). Hopefully, checkpoint-based therapy was effective and beneficial against advanced HCC clinically (18, 19). However, owing to the low sensitivity and unexpected resistance to ICIs, more useful and reliable biomarkers should be identified to improve the accuracy of predicting the prognosis and immunotherapy efficiency in HCC (20). How to choose available and suitable targets for personalized therapy is still a tricky question to be answered for HCC patients.

TME is a highly heterogeneous ecosystem involving different types of stromal cells, vascular cells, and immune cells perturbed by therapy, which are recognized as the potential determinants of treatment response in cancer (21, 22). Tumor immunological phenotype (TIP) is an emerging concept to evaluate the immunological heterogeneity depending on the relative infiltration of immune cells (23), and tumors are generally classified into two TIPs: “hot” (inflamed) and “cold” (non-inflamed) (23). Particular genes and pathways genetically regulate the immunological phenotypes have been identified to aid immunotherapy (24–27). Wang et al. recently reported 12 hot tumor-related genes and three cold tumor-related genes to constitute the TIP gene signature using a text-mining approach (26), which is significantly associated with the survival outcomes of cancer patients and shows superior performance in predicting immunotherapeutic responses than widely used immune signatures such as tumor mutation burden (TMB), and tumor immune dysfunction and exclusion (TIDE). Thus, these “TIP genes” hold great promise in clinical application especially for postoperative risk stratification and the discovery of immunotherapeutic predictors.

**Figure 1:**
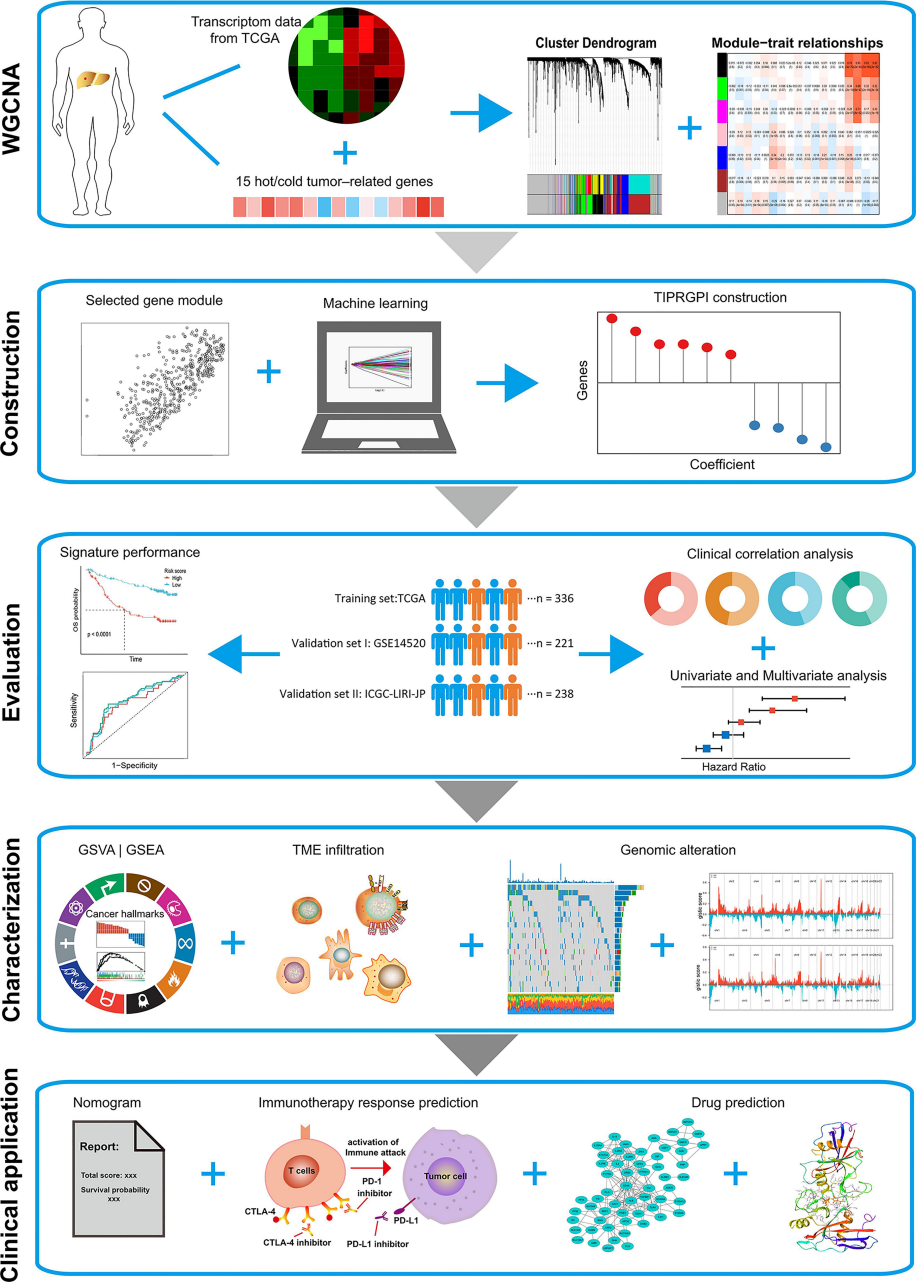
Flowchart of this study.

Accumulating immune-based signatures have been established to predict HCC patients’ outcomes. However, the predictive accuracies of most signatures are still insufficient for clinical practice and a more reliable and accurate signature predicting the survival as well as the immunotherapy response of HCC patients is urgently needed (28, 29). In this study, a “TIP genes”-guided strategy was employed with several statistical algorithms to construct TIP-related gene prognostic index (TIPRGPI), a novel HCC signature, followed by comprehensive validation to predict the prognosis and immunotherapy efficiency for HCC patients. Besides, it is estimated that the low-risk group might respond better to immunotherapies than those in the high-risk group. Furthermore, we identified six potential drugs binding well to the core target of TIPRGPI with molecular docking. The workflow for this study is shown in **Figure 1**.

## Materials and Methods

### Data Source

We carefully reviewed the mRNA expression datasets deposited in the Cancer Genome Atlas (TCGA), International Cancer Genome Consortium (ICGC), and Gene Expression Omnibus (GEO), database and enrolled the patients with complete annotation of overall survival (OS).Those patients with an overall survival time of <30 days were excluded due to other possible causes of mortality (29). Subsequently, the RNA-seq gene expression data of 336 HCC patients was derived from the TCGA database (https://portal.gdc.cancer.gov/) (TCGA-LIHC). The corresponding clinical information and survival outcomes including overall survival, progression-free survival (PFS), disease-specific survival (DSS) and disease-free survival (DFS) were also collected. Another RNA-seq expression profiling dataset (ICGC-LIRI-JP) containing 238 patients with survival information was obtained from the ICGC database (https://dcc.icgc.org). In addition, we acquired a transcriptomic microarray dataset (GSE14520) including a total of 221 HCC patients (30, 31) from the GEO database (https://www.ncbi.nlm.nih.gov/geo/). The summary of the demographic information was listed in **Supplementary Table 1**. For the TCGA-LIHC dataset, the Fragments per Kilobase Million (FPKM) value was used to generate the Transcripts per Kilobase Million (TPM) and further subjected to log2 transformation for normalization. For the ICGC-LIRI-JP and GSE14520 datasets, data were preprocessed as previously reported (32, 33). The ESTIMATE algorithm was utilized to calculate the immune score, stromal score, estimate score, and tumor purity for all the patients in the TCGA-LIHC dataset (34, 35).

Somatic mutation information of TCGA-LIHC was gathered from the TCGA data portal (http://tcga-data.nci.nih.gov/tcga/) as the mutation annotation format (MAF) format by the R package “maftools” (36). The CNV profile contained in the “Masked Copy Number Segment” data type was downloaded from TCGA.

### Correlations of TIP Score With Prognosis and TME of HCC

TIP score was calculated as previously reported (26) with some modifications. Briefly, the gene expression matrix of three predefined cold tumor-related genes (CXCL1, CXCL2, and CCL20) and 12 predefined hot tumor-related genes (CXCR3, CXCR4, CXCL9, CXCL10, CXCL11, CCL5, CD3, CD4, CD8a, CD8b, CD274, and PDCD1) was extracted, followed by the generation of expression z scores. TIP score was computed by a summary score of RNA-seq z scores for the tumor immunological phenotype genes. To evaluate the prognostic value of the TIP score, all patients with available survival information for OS, DFS, PFS, and DSS were divided into the high- and low-score group by the optimal cutoff of TIP scores, respectively (34), followed by the Kaplan-Meier analysis with a log-rank test. To examine the relationship between TIP score and TME, we carried out the Spearman correlation analysis between TIP score and the ESTIMATE derived scores including immune score, stromal score, estimate score, and tumor purity. And we also checked the correlations of the TIP score and the fractions of the activated CD4 and the activated CD8, as well as two immune checkpoint molecules (PD1 and CTLA-4).

### Weighted Gene Co-Expression Network Analysis (WGCNA) and TIPRGPI Establishment

WGCNA was performed on the expression data of TCGA-LIHC using the “WGCNA” R package (37). Generally, all genes were sorted by the median absolute deviation (MAD), and the top 5,000 genes were used for sample clustering, followed by the removal of outlier samples. Then, the optimal soft threshold power was specified to generate a scale-free network. Next, the topological overlap matrix (TOM)-based dissimilarity (dissTOM) was computed and further used to perform the gene dendrogram and module recognition with the minClusterSize of 30. Similar dynamic modules were merged by the cutline of 0.2. Pearson correlation coefficients (PCC) and corresponding P values between module eigengenes and clinicopathological parameters were subsequently calculated and visualized by a heatmap. The most significant module that correlated with the TIP score was identified and used for further analysis.

Gene Ontology (GO) enrichment and Kyoto Encyclopedia of Genes and Genomes (KEGG) pathway analysis were completed for the most significant module by the “clusterProfiler” R package (38) with the cutoff of p.adjust <0.05.

To establish a scoring system regarding TIP score, we first adopted the Univariate Cox (UniCox) hazard regression to screen the candidate genes from the most significant module. Next, the popular least absolute shrinkage and selection operator (LASSO) algorithm was applied for the best subset of prognostic genes using the “glmnet” R package (39). For the purpose of minimization of the overfitting risk, we conducted LASSO 200 times and chose the robust genes that appeared in the model more than 160 times. A linear equation called “TIPRGPI” was then established to predict the overall survival of HCC patients: Risk score = Σ(coef (β)*EXP_β_), where β represents each selected gene.

### Survival Analysis

The TIPRGPI score was calculated for each patient of the TCGA-LIHC training set, the ICGC-LIRI-JP validation set, and the GSE14520 validation set. For each dataset, patients were separated into the high- and low-risk groups by the median value of the training set, which is crucial for clinical practice. Kaplan-Meier survival curves were depicted to compare the difference of distinct risk groups with a log-rank test, and time-dependent receiver operating characteristic (tROC) curves were drawn to assess the predictive power. Moreover, stratified analysis was performed to further validate the additional prognostic value of the TIPRGPI model, and univariate and multivariate analyses were used to determine the independent prognostic indicators for HCC. Additionally, we also compared the 3- and 5-year ROC values of the TIPRGPI and popular biomarkers for immunotherapy and other published gene signatures of HCC, including a TP53-related transcriptomic signature by Long et al. (“Long signature”) (40), a metabolic gene signature by Huo et al. (“Huo signature”) (27), a ferroptosis-related gene signature (“Liang signature”) (41), an immune-related prognostic signature (“Wang signature”) (42), and a hypoxia-related risk signature by Zeng et al. (“Zeng signature”) (43).

### Construction of a Predictive Nomogram

A TIPRGPI-integrated nomogram was constructed to quantitatively evaluate the prognostic risk based on the result of univariate analysis. Calibration curves for the 3- and 5-year were drawn to examine the predictive capability of the nomogram. The 1-, 3-, and 5-year DCA plots were utilized to measure the net benefits of the nomogram and TNM stage, as well as tumor burden. Moreover, Kaplan-Meier analysis was further used for OS, DFS, PFS, and DSS on the TCGA-LIHC set to validate the prognostic value of the nomogram.

### Genomic Variation Analysis

To explore the somatic mutations regarding TIPRGPI, the “maftools” R package was used to depict the waterfall plots manifesting the mutation landscape for the high- and low-risk groups of HCC patients. TMB values reflecting total mutation numbers for each HCC patient were calculated with non-synonymous mutations using 38MB as the estimate of the exome size (44, 45). Somatic copy number alterations between the two different risk groups were investigated *via* the GISTIC2.0 algorithm. The correlations of expression values and CNV types for two oncogenic hub genes (NDC80 and RFC4) (32, 33) in HCC were accomplished by the Kruskal-Wallis test.

### Gene Set Variation Analysis (GSVA) and Gene Set Enrichment Analysis (GSEA)

To determine the underlying hallmark pathways related to TIPRGPI, the R package “GSVA” was utilized to obtain the GSVA enrichment scores (46) of the 50 hallmark pathways (h.all.v7.1.symbols) deposited in the molecular signature database (47, 48) for each patient in the high- and low-risk groups of the TCGA-LIHC dataset, followed by the contrast of GSVA scores using a linear model as previously reported (49). Significant gene sets were defined by an adj.*P*.Val of < 0.01. GSEA for the same 50 hallmark gene sets was operated in the two risk groups with the recommended criteria of FDR<0.25 and NES>1. Venn diagram analysis was performed to identify the overlapping hallmark pathways by GSVA and GSEA, and Kaplan-Meier analysis was further used to verify the prognostic value of oncogenic hallmark pathways.

### Exploration of Immune Infiltration

To investigate the relative infiltration of TME cells in the high- and low-risk groups of HCC, the ssGSEA algorithm was utilized for immune deconvolution analyses with the gene sets of 28 reported immune cell types (50) and two stromal components (fibroblasts and endothelial cells) (51) of TME. Differential infiltration analysis was conducted and visualized by a violin plot and the relationship of the TIPRGPI score and each type of the 30 TME cells was determined by the Spearman correlation analysis. Kaplan-Meier survival analysis was also performed to assess the prognostic values of these TME cells.

### Estimation of Immunotherapeutic Response Prediction

According to previous publications, the correlations between TIPRGPI and potential immunotherapeutic markers including 50 ICB-related genes (52–54), IFN-gamma pathway markers (55), and m6A regulators (56, 57) were explored by Wilcoxon test. The Tumor Immune Dysfunction and Exclusion (TIDE) algorithm (58) (http://tide.dfci.harvard.edu/), was utilized to infer the clinical response to immunotherapy with the gene expression profile of TCGA-LIHC. Additionally, Immunophenoscore (IPS), which was designed to determine immunogenicity using the machine learning approach, was further obtained from The Cancer Immunome Atlas (TCIA) (https://tcia.at/home) (50). Higher IPS indicates a better response to immunotherapy.

### Identification of the Core Target of TIPRGPI

To identify the core target of the TIPRGPI signature, all genes were uploaded to the online database of the Search Tool for the Retrieval of Interacting Genes (STRING) (version 11.0; http://string-db.org/) for the construction of the protein-protein interaction (PPI) network with default settings (Interaction score ≥0.4). Cytoscape (version 3.2.1; http://www.cytoscape.org) was used for visualization. Next, we calculated the topological parameters with the Network Analyzer plugin and obtained the degrees of all nodes in the network. The core target was recognized as the node with the highest degree.

### Molecular Docking

For the screening of the putative small molecules stably binding to the core target, molecular docking was performed with Glide of Schrodinger. Firstly, we collected the 3D protein structures of totally 9800 small molecules as well as the core target from zinc15 database and PDB database (www.rcsb.org), respectively. Next, the protein preparation wizard tool was utilized to process the crystal structure. Subsequently, the ligand-binding pocket was predicted with the deepsite module (59) of Play Molecule (www.playmolecule.org), which was a knowledge-based approach using convolutional neural networks. Finally, the binding mode and interaction force of the core target and small molecules were evaluated to identify potential compounds.

### Statistical Analysis

The correlations of TIP score and immune signatures were conducted using Spearman correlation by the “ggplot2” package. Kaplan-Meier analysis was performed using the “survival” package with a log-rank test. The correlations of the TIPRGPI group and other clinicopathological features were determined by the Pearson Chi-square test. Univariate and multivariate analyses were applied by the “survival” package to identify independent prognostic indicators. The optimal cutoff for survival analysis was generated by the R package “survminer”. All statistical analyses were completed by the R software (version 3.6.1). Unless specified otherwise, *P* < 0.05 was considered statistically significant.

## Results

### TIP Score Was Associated With the Prognosis and the Immune State of HCC

**Figure 2:**
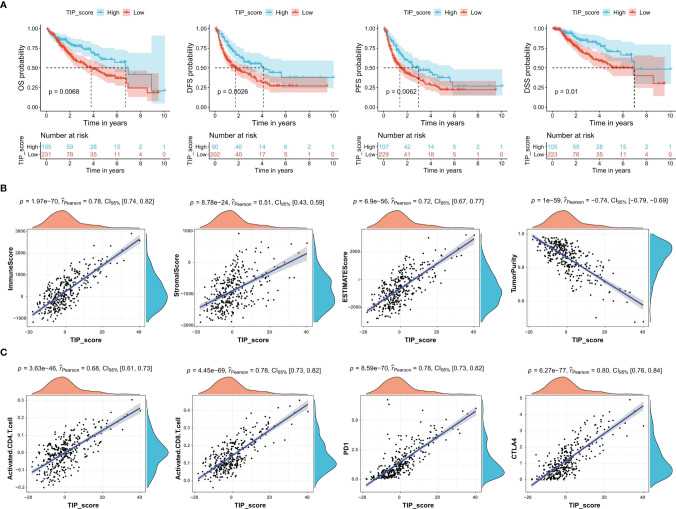
TIP score correlates with the prognosis and the immune state of HCC. **(A)** Kaplan–Meier survival plots of TIP score for OS, DFS, PFS, and DSS in TCGA-HCC cohort. **(B)** Correlations between TIP score and immune score, stromal score, estimate score, and tumor purity in HCC. **(C)** Correlations between TIP score and biomarkers of cancer immunotherapy including activated CD4/CD8 and PD-1/CTLA-4. TIP, tumor immunological phenotype. OS, overall survival; DFS, disease-free survival; PFS, progression-free survival; DSS, disease-specific survival.

To determine whether TIP score was effective in HCC, we carried out a series of survival analyses, applying Kaplan-Meier (K-M) survival curves and log-rank tests to investigate the discrepancy between low- and high- TIP score groups. As expected, patients with HCC in the high TIP score group had a better prognosis (**Figure 2A**). Next, we confirmed the correlations between TIP score and the immune score, stromal score, estimate score, and tumor purity respectively. As shown in **Figure 2B**, TIP score was positively associated with immune score, stromal score, and estimate score, but negatively associated with tumor purity. Moreover, given that effective T cells such as activated CD4 and CD8 T cells play a pivotal role in the tumor microenvironment (60), we also calculated their correlations with TIP score, and we found they were both correlated with TIP score positively (**Figure 2C**). Besides, considering that PD-1 or CTLA-4 is the key immune checkpoint, we also verified they were positively correlated with TIP score (**Figure 2C**).

### Construction of TIPRGPI

**Figure 3:**
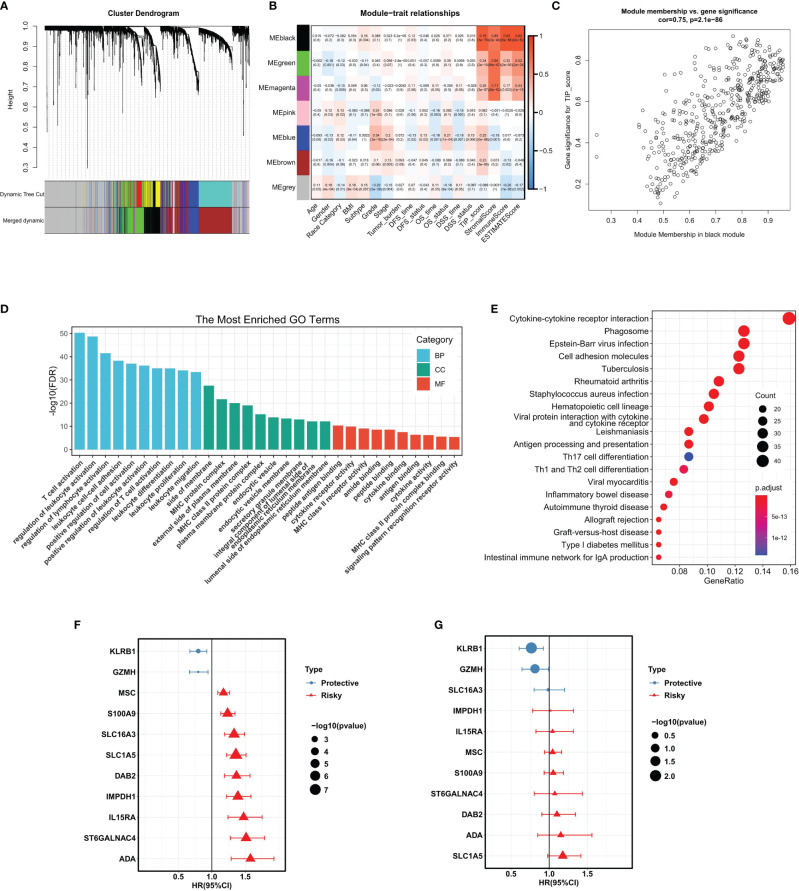
WGCNA analysis and the construction of TIPRGPI for HCC. **(A)** Cluster dendrogram of MAD top 5000 genes. **(B)** Table cells showing Pearson’s correlation coefficients and corresponding *P*-value between module eigengenes (ME) and the variables. **(C)** Scatter plot showing the relationship between gene significance (GS) for TIP score and module membership (MM) in the black module. **(D, E)** GO enrichment analysis **(D)** and KEGG **(E)** enrichment analysis for the black module genes. **(F, G)** Hazard ratio with 95%CI of each gene in the TIPRGPI signature computed by UniCox and MultiCox, respectively. GO, gene oncology; KEGG, Kyoto Encyclopedia of Genes and Genomes; WGCNA, weighted gene co-expression network analysis; MAD, median absolute deviation; TIPRGPI, TIP-related gene prognostic index.

In order to identify the gene module associated with TIP score, WGCNA was applied to the TCGA-LIHC RNA-seq dataset. The MAD top 5000 genes were extracted to construct a co-expression network. Four outlier samples were removed prior to network construction (**Supplementary Figure 1A**). The optimal soft-thresholding power of 10 (scale-free R^2^ = 0.86) was picked to ensure the scale-free topology (**Supplementary Figure 1B**). The established co-expression network showed that these 5000 genes were clustered into seven modules (**Figure 3A**) and the network heatmap plot of the clustering dendrogram among modules was shown (**Supplementary Figure 1C**). Then, we calculated the correlations of module eigengenes (ME) with multiple indicated variables by computing the Pearson correlation coefficient (PCC), and among which, we focused on the black module showing highly positive correlation with TIP score (PCC = 0.78, *P* = 3E-70) (**Figure 3B**). Also, we plotted a scatterplot of gene significance vs. module membership of the black module containing 432 genes (**Figure 3C**). GO enrichment analysis revealed that the most significant terms enriched by the black module were the biological process (BP) of T cell activation, cellular component (CC) of side of membrane, and molecular function (MF) of peptide antigen binding (**Figure 3D**). KEGG analysis suggested that they mostly participated in the pathway of cytokine-cytokine receptor interaction (**Figure 3E**). Subsequently, we inputted the genes of the black module into UniCox regression analysis and found 128 significant genes with *P* value lower than 0.05 (**Supplementary Table 2**). Next, we conducted LASSO Cox regression with the 128 genes and obtained 11 robust genes (KLRB1, GZMH, SLC16A3, IMPDH1, IL15RA, MSC, S100A9, ST6GALNAC4, DAB2, ADA, SLC1A5) that were significantly correlated with the OS of HCC patients from TCGA-HCC dataset. MultiCox was applied to analyze the 11 genes, which were subsequently incorporated into a TIPRGPI model for predicting the prognosis of HCC. **Figures 3F, G** showed the UniCox and MultiCox results of the selected 11 genes with the corresponding hazard ratio (HR) and statistical significance.

### Evaluation and Validation of the TIPRGPI Signature

**Figure 4:**
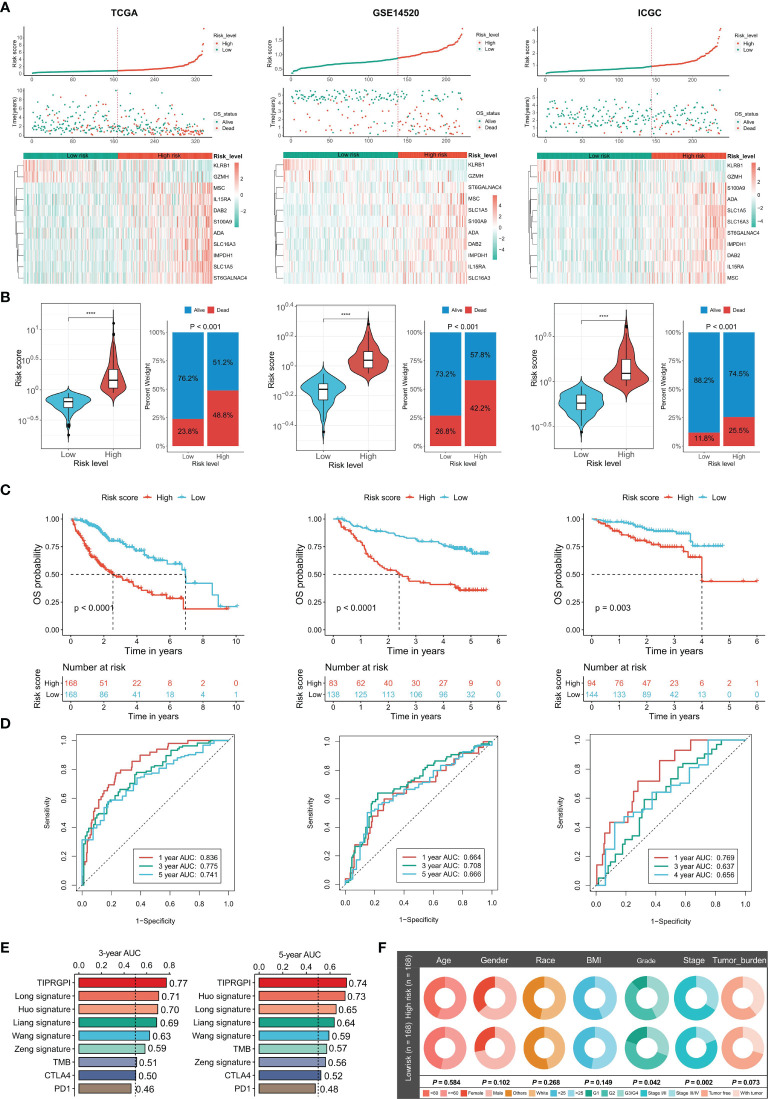
Validation of the TIPRGPI predicting model and performance analysis in HCC. **(A)** Risk score distribution, survival status, and the expression of 11 TIPRGPI genes for patients in low- and high-risk groups from TCGA training dataset and two validation datasets (GSE14520 and ICGC-LIRI-JP). **(B)** Risk score and mortality rate of patients in low- and high-risk groups from three datasets. **(C)** Kaplan-Meier survival curves showing the comparison of overall survival (OS) between the low- and high-risk groups from three datasets. **(D)** Time-dependent receiver operating characteristic (tROC) curves of three datasets. **(E)** The area under the ROC curve (AUC) in 3-year and 5-year survival for TIPRGPI and other published signatures and common immunotherapeutic biomarkers. **(F)** Correlation analysis between the TIPRGPI low-/high-risk groups and clinical traits ****P < 0.0001.

After the construction of TIPRGPI, we proceeded with evaluation and validation analysis. First, we computed the risk score for individual patients using the expression and the risk coefficients of the 11 TIPRGPI genes in HCC datasets, and based on the median value derived from the training set, HCC patients from TCGA-LIHC (training dataset), GSE14520 (validation dataset 1) and ICGC-LIRI-JP (validation dataset 2) were separated into low- and high-risk groups, respectively (**Figure 4A**). As shown in **Figure 4B**, the low-risk group had a lower death rate than the high-risk group. Afterward, by Kaplan-Meier analysis, significant differences in the OS possibility were observed between the low- and high-risk groups in the training and validation datasets (**Figure 4C**). Further, the time-dependent receiver operating characteristic curve analysis was applied to evaluate the accuracy of the TIPRGPI signature. For the TCGA-HCC training dataset, the area under the ROC curve (AUC) was 0.836, 0.775, and 0.741 in 1-year, 3-year, and 5-year survival, respectively. Moreover, ROC curve analysis of GSE14520 and ICGC validation dataset exhibited that TIPRGPI had excellent predictive performance (GSE14520: AUC = 0.664 for 1-year, 0.708 for 3-years and 0.666 for 5-year survival; ICGC: AUC = 0.769 for 1-year, 0.637 for 3-years and 0.656 for 4-year survival) (**Figure 4D**). Compared with several other published signatures and popular biomarkers, TIPRGPI had the highest AUC for either 3-year or 5-year survival (**Figure 4E**). Besides, stratified analysis revealed an additional predictive value of TIPRGPI in subgroups divided by age, gender, BMI, race, stage, grade, and tumor burden (**Supplementary Figure 2**). Correlation analysis between TIPRGPI and multiple clinical traits revealed that the tumor grade and stage of HCC were significantly correlated with risk score (**Figure 4F**). These results indicated that TIPRGPI was a highly reliable signature.

### Establishment of the Prognostic Nomogram

**Figure 5:**
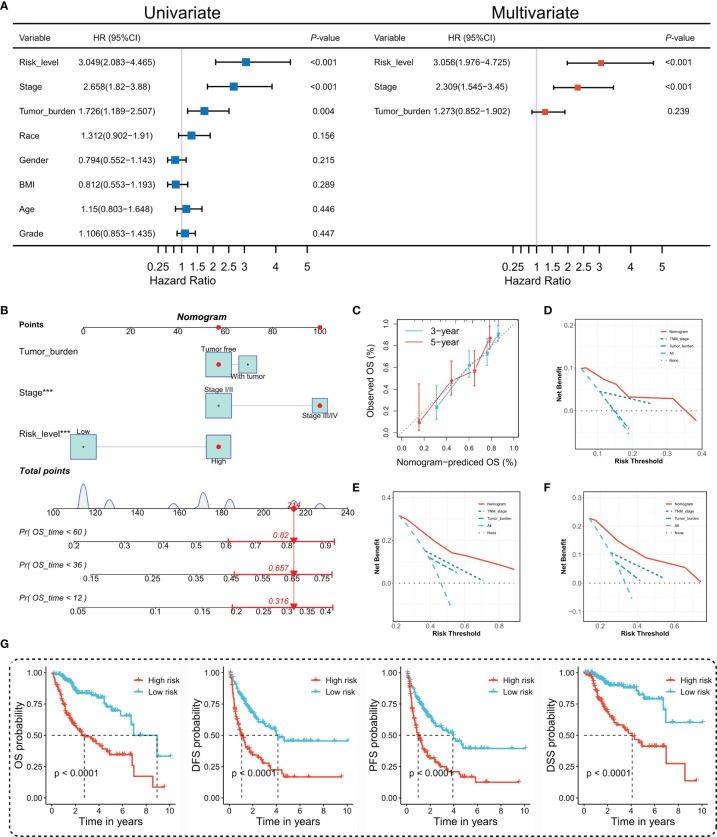
Construction and evaluation of the TIPRGPI-integrated nomogram. **(A)** Univariate and multivariate analyses of the clinical traits and TIPRGPI for the OS. **(B)** Nomogram for predicting the probability of 1-, 3-, and 5-year overall survival in HCC. **(C)** The calibration plots of the nomogram predicting the probability of the 3- and 5-year OS. **(D–F)** Decision curves showing the comparison of net benefits of the nomogram, TNM stage, tumor burden for 1-year **(D)**, 3-year **(E)**, and 5-year **(F)** OS. **(G)** Kaplan-Meier survival analysis of the integrated nomogram for OS, DFS, PFS, and DSS of HCC. ***P < 0.001.

To figure out whether the TIPRGPI predicting model was an independent prognostic indicator in HCC, univariate and multivariate analyses were performed. The HR of the TIPRGPI risk level was 3.049 (95%CI: 2.083-4.465) and 3.056 (95%CI: 1.976-4.725) in the univariate and multivariate analysis, respectively, and an elevated HR was observed compared with the pathologic stage (**Figure 5A**). Importantly, multivariate analysis demonstrated TIPRGPI was an independent prognostic factor in HCC.

To provide a quantitative instrument for the clinician, a nomogram was built by tumor burden, stage, and TIPRGPI (**Figure 5B**). The calibration plot showing the observed versus predicted rates of the 3- and 5-year OS indicates the ideal consistency of the nomogram (**Figure 5C**). As **Figures 5D–F** showed, the TIPRGPI-integrated nomogram achieved a better net benefit than clinical traits in predicting 1-year, 3-year, and 5-year OS in HCC patients from the TCGA-LIIHC dataset. In addition, we confirmed the prognostic value of the nomogram, which was found to be significantly associated with OS, DFS, PFS, and DSS, respectively (**Figure 5G**).

### The Underlying Molecular Mechanisms of TIPRGPI

**Figure 6:**
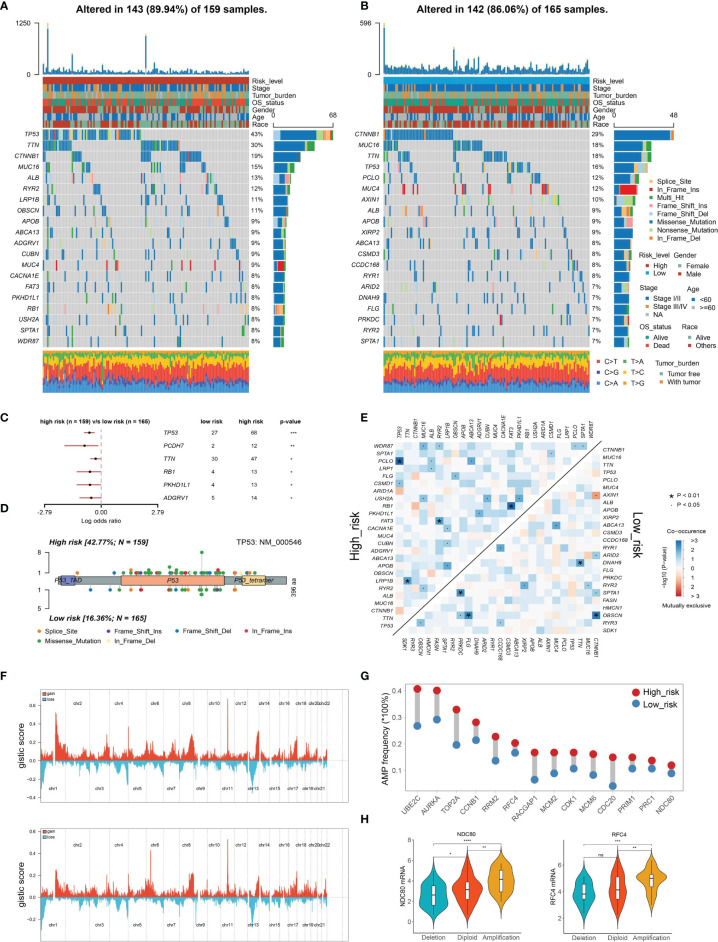
Genetic alterations of the TIPRGPI low- and high-risk groups. **(A, B)** Waterfall plots displaying the mutation landscapes of the high- **(A)** and low-risk groups **(B)**. **(C)** Forest plot showing the significantly different mutated genes between the TIPRGPI risk groups. **(D)** Lollipop plot indicating the distribution of mutation spots in the high- and low-risk groups. **(E)** The coincident and exclusive associations across the top mutated genes in high- and low-risk groups. **(F)** The distribution of CNV features across all chromosomes for the high- (upper) and low- (bottom) risk groups. **(G)** HCC hub genes were widely amplified in the high-risk group. **(H)** Violin plots indicating the positive correlation of gene expression and copy number of two represented hub genes (NDC80 and RFC4) in the high-risk group. *P < 0.05, **P < 0.01, ***P < 0.001, ****P < 0.0001, ns, not significant.

To investigate the potential mechanisms of the risk level defined by TIPRGPI in HCC, we downloaded the available somatic mutation profiles and analyzed the mutation landscape of the high- and low-risk patients from the TCGA-LIHC dataset (**Figures 6A, B**). We exhibited the top 20 mutated genes in two groups respectively. The gene with the most mutation frequency is TP53 (43%) in the high-risk group and that in the low-risk group is CTNNB1 (29%). The summary of the mutation information with statistical calculation was shown in **Supplementary Figure 3**. Further, the significant differentially mutated genes between the TIPRGPI high- and low-risk groups were detected by Fisher’s exact test. As shown in **Figure 6C**, TP53 was found with a much higher mutation rate in the high-risk group compared with the low-risk group (*P* < 0.001), and a lollipop plot was depicted to indicate the different mutation spots of TP53 for the two risk groups (**Figure 6D**). Meanwhile, the coincident and exclusive associations across the top 25 mutated genes from the high- and low-risk groups were also analyzed, in which blue represents the co-occurrence while red represents mutual exclusion (**Figure 6E**). Additionally, the CNV alteration landscapes of the high- and low-risk groups were generated after removing the germline features (**Figure 6F**). Interestingly, the hub genes of HCC (32, 33) were widely amplified in the high-risk group in comparison with the low-risk group (**Figure 6G**). NDC80 and RFC4 were two examples demonstrating the positive correlations of gene expression and copy number in the TIPRGPI high-risk group (**Figure 6H**).

**Figure 7:**
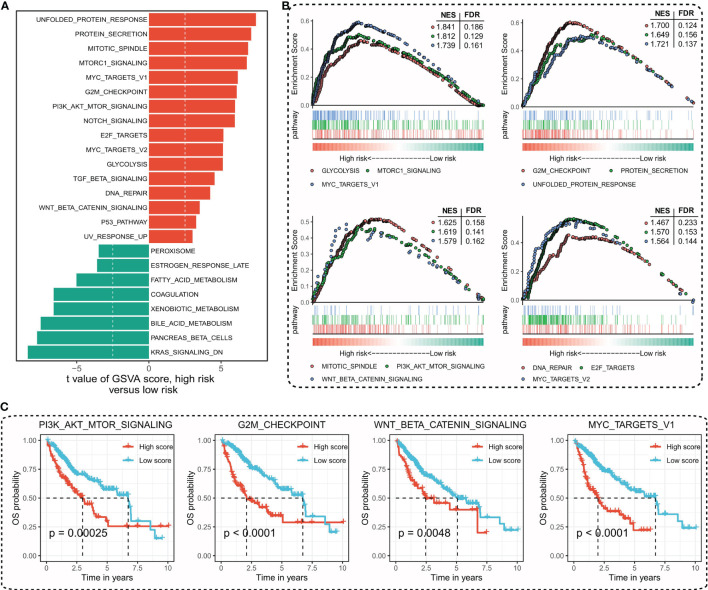
Determination of the distinct hallmark pathways of the TIPRGPI low- and high-risk groups. **(A)** Differences in cancer hallmark pathway activities between the high- and low-risk groups scored by GSVA. **(B)** The GSEA results for the 12 overlapping upregulated hallmark pathways in terms of the TIPRGPI risk groups. **(C)** Kaplan-Meier survival plots showing the significant correlations between the OS and GSVA scores of typical oncogenic hallmark pathways. GSVA, gene set variation analysis. GSEA, gene set enrichment analysis.

To explore the associated cancer hallmark pathways regarding TIPRGPI, we performed GSVA in high- and low-risk groups. According to the predefined cutoff, 16 hallmark pathways significantly increased in the high-risk group compared with the low-risk group (**Figure 7A**). GSEA confirmed that 12 of them were upregulated in the high-risk group, most of which were related to well-known oncogenic pathways (61) (**Figure 7B** and **Supplementary Table 3**). Kaplan-Meier survival analysis was applied to evaluate the prognostic values of the upregulated hallmark pathways and different OS probabilities were observed between the high- and low-score groups for these oncogenic hallmark pathways, such as PI3K_AKT_MTOR_SIGNALING, G2M_CHECKPOINT, WNT_BETA_CATENIN_SIGNALING, and MYC_TARGETS_V1 (**Figure 7C**). Taken together, TIPRGPI was tightly associated with oncogenic pathways.

### TIPRGPI Was Associated With HCC Immune Status

**Figure 8:**
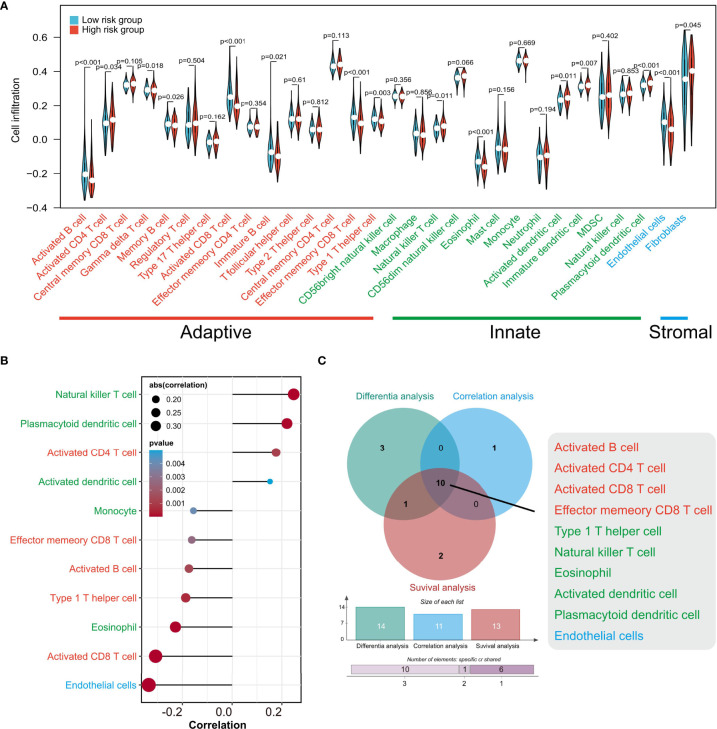
The associations of TIPRGPI and infiltration of TME cells estimated by ssGSEA. **(A)** The differences of TME cell infiltration between low- and high-risk groups. **(B)** Correlations between the risk score and TME cells. **(C)** Venn diagram revealing 10 types of most relevant TME cells contributing to the risk stratification of HCC patients by TIPRGPI. TME, tumor microenvironment.

Given that TIPRGPI was constructed on the basis of TIP score, which was significantly related to other immune signatures, we explored the potential relationship between TIPRGPI and the infiltration of TME cells. The boxplots showed the differential distribution of infiltrating TME cells inferred by ssGSEA algorithm between low- and high-risk groups and revealed that the infiltration of most TME cell types including activated B cell, activated CD4 T cell, gamma delta T cell, memory B cell, activated CD8 T cell, immature B cell, effector memory CD8 T cell, type 1 T helper cell, natural killer T cell, eosinophil, activated dendritic cell, immature dendritic cell, plasmacytoid dendritic cell, endothelial cell and fibroblast cell (*P* < 0.05) were significantly associated with risk group (**Figure 8A**). In addition, correlation analysis was used to pick out the TME cell types significantly correlated with the risk score and the result showed that four types were positively correlated with risk score while seven types were negatively correlated with it (**Figure 8B**). We also analyzed the relationships between OS and the infiltration of TME cells, whose expression levels were classed into low- and high-infiltration groups from the TCGA-HCC dataset, and the results showed 13 TME cell types were involved in the significant differences between the high- and low-infiltration groups (**Supplementary Figure 4**). Finally, overlapping Venn plot revealed 10 intersected TME cell types including four adaptive immune cell types (red), five innate immune cell types (green), and one stromal cell type (blue) among differential analysis, correlation analysis, and survival analysis (**Figure 8C**). These findings strongly suggested that the infiltration of TME cells plays a vital role in the postoperative risk stratification of HCC.

### TIPRGPI May be a Potential Indicator to Predict Immunotherapeutic Sensitivity in HCC Patients

**Figure 9:**
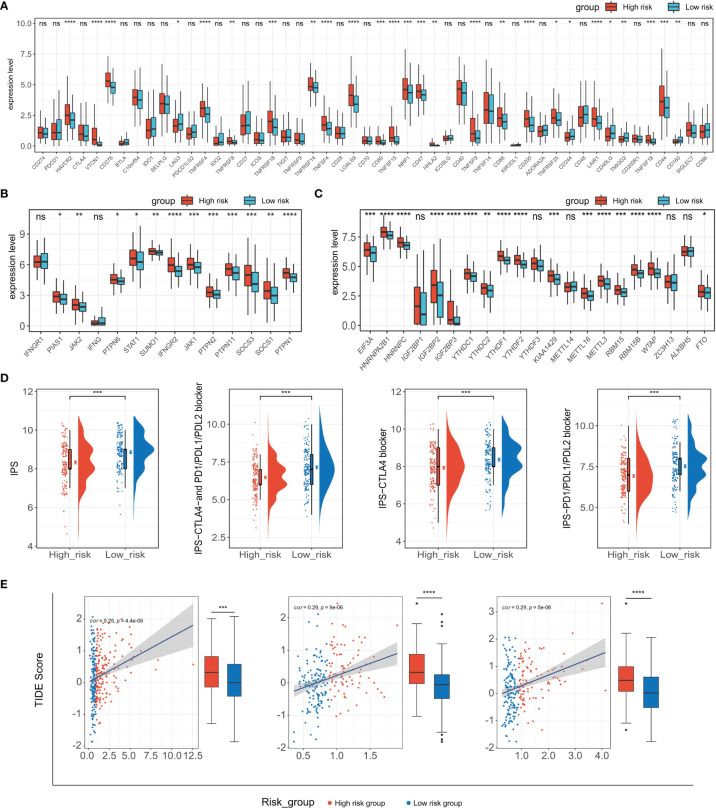
Application of the TIPRGPI model for immunotherapy prediction. **(A–C)** Expression of immune checkpoint molecules **(A)**, IFN-gamma pathway-related markers **(B)**, and m6A regulators **(C)** in low- and high-risk groups. **(D)** The relationship between TIPRGPI and IPS. **(E)** Distribution of TIDE scores in the TCGA-LIHC, GSE14520, and ICGC-LIRI-JP datasets. *P < 0.05, **P < 0.01, ***P < 0.001, ****P < 0.0001, ns, not significant.

ICB-related gene expression levels have been reported to be correlated with therapeutic responses of immune checkpoint inhibitors (62) and ICB targeting promising checkpoints has emerged as a promising strategy in treating cancers (63). To evaluate the potential of TIPRGPI for predicting the response of HCC patients to immunotherapy, we first determined the expression of 50 immunomodulators in low- and high-risk groups. As shown in **Figure 9A**, the expressions of more than half of the presented immunomodulators were significantly associated with the risk score. Considering the significant correlation between the risk score and CD8 T cell and the important role of m6A methylation in impairing the anti-tumor ability of CD8 T cell, we next analyzed the expression of the CD8 T cell-related IFN-gamma pathway markers and m6A regulators in low- and high-risk groups and found most of them were significantly associated with the risk score (**Figures 9B, C**). These findings demonstrated the TIPRGPI had great potential in evaluating the response of immunotherapy for HCC. Subsequently, we explored the correlation between the TIPRGPI risk group and immunophenoscore (IPS), which is a recognized model based on machine learning to predict patients’ responses to immune checkpoints blockade by estimating the immunogenicity. We found that the low-risk group has a higher IPS score, indicating patients in the low-risk group might respond better to immunotherapy (**Figure 9D**). We also used the TIDE algorithm to predict the immunotherapeutic efficacy for immune checkpoint blockade in TCGA-LIHC, GSE14520, and ICGC datasets and the high-risk group had a higher TIDE score, indicating that high-risk patients might have a worse response to immunotherapy (**Figure 9E**).

### Core Target Identification and Candidate Molecules Prediction

To identify the core target concerning TIPRGPI, a PPI network was built using the STRING database (confidence score > 0.4) and visualized by the Cytoscape software (**Supplementary Figure 5**). The highly interactive network contains 58 nodes and 182 edges with the clustering coefficient of 0.595. As shown in **Supplementary Figure 5**, CD44 lies at the hub of the network and has the highest degree among all the nodes. Thus, CD44 was considered as the core target.

**Figure 10:**
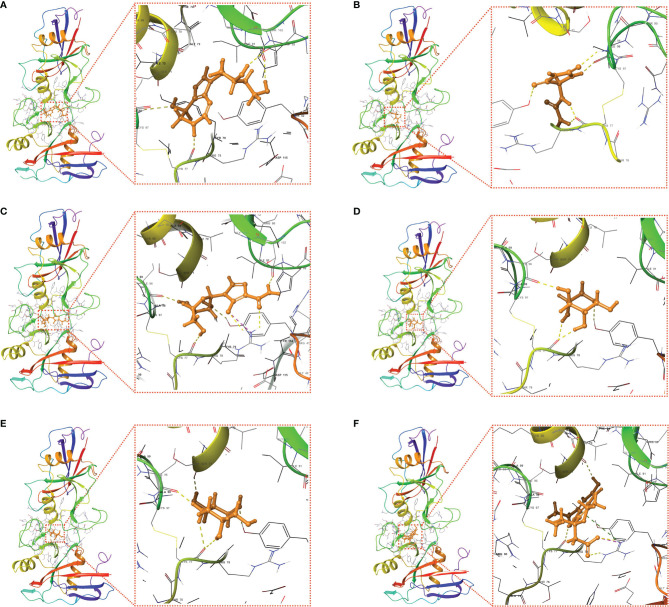
Putative docking models of the six candidate drugs and the core target using molecular docking analysis. 3D structures and binding modes showing the formed hydrogen bonds between the predicted pocket of CD44 and Pentostatin **(A)**, Allantoin **(B)**, Mizoribine **(C)**, Xylose **(D)**, Deoxynojirimycin **(E)**, and 6-Hydroxyetodolac **(F)**.

Molecular docking is a structure-based computational algorithm for compound screening. In this study, the structures for a total of 9800 purchasable small molecules from the libraries of zinc15 database were obtained and subjected to molecular docking. **Supplementary Table 4** showed the top six molecules (Pentostatin, Allantoin, Mizoribine, Xylose, Deoxynojirimycin, and 6-Hydroxyetodolac) that had the highest affinity with the predicted binding pocket of CD44. The 3D diagrams for the six docking models presenting the detailed binding energy were displayed in **Figure 10**. For instance, Mizoribine (ZINC000003812887) forms hydrogen bonds with amino acid residues ILE-96, CYS-77, ARG-78, TYR-42, and ILE-91. Besides, the probable formation of salt bridge interaction between ARG-78 and the side-chain hydroxyl group of the ligand also helped the compound connect to the active site of CD44.

## Discussions

It is well established that the tumor immune microenvironment is closely related to tumorigenesis and cancer progression (64–66). Here, using the “TIP genes”-guided strategy with multiple statistical approaches, we developed a novel immune-relevant and independent predictive model - TIPRGPI - for prognosis and immunotherapy in HCC. Based on the training (TCGA-LIHC) and two external validation (ICGC-LIRI-JP and GSE14520) datasets, the TIPRGPI signature was applied to divide HCC patients into low-risk and high-risk groups. As expected, high-risk patients had a worse prognosis and response to immunotherapy. Univariate and multivariate analysis verified it was an independent predictor for the prognosis of HCC. Moreover, a TIPRGPI-integrated nomogram model was established, which showed a better net benefit than the clinical traits in predicting 1-year, 3-year, and 5-year OS for HCC patients, thus demonstrating enhanced accuracy and potential implication in clinical practice. Besides, potential drugs targeting the signature could be predicted *via* molecular docking. Therefore, the TIPRGPI signature was a reliable model to predict prognosis and immunotherapeutic response in HCC and might provide valuable insight for seeking the treatment for HCC.

A growing body of gene signatures has been established to shed light on the prognosis classification of HCC. For example, Long et al. established a four-gene prognostic model that showed a good performance for HCC prognosis prediction (40). Gao et al. reported a six-gene signature for predicting OS of HCC (67). However, few studies focused on gene signatures on the basis of key genes in the tumor immune microenvironment. Tumor immunological phenotype has been emerging to be significantly related to prognosis and therapeutic responses in various types of cancer by mounting evidence (26, 68–71). TIP score, representing the expression level of fifteen TIP genes that were obtained from a previously study (26), was significantly associated with the prognosis and other immune signatures in HCC. Thus, we, for the first time, conducted WGCNA to uncover the specific gene expression pattern related to TIP score in HCC, identifying the black module that contained 432 genes, which generated an 11-gene signature called “TIPRGPI” for prognosis prediction of HCC. Another advantage of this study is the larger sample size and higher AUCs than that of most previous studies trying to building an effective risk classifier for HCC. Besides, unlike previous studies only describing a prognostic gene signature, we engaged an integrative analysis to get a deeper and comprehensive understanding of the risk model, and putative drugs were even predicted based on the model, which was rarely reported by other similar studies.

All genes involved are either related to the immune system or tumorigenesis. For example, adenosine deaminase (ADA) is produced in all cells but highest in lymphocytes and loss of ADA causes the immune system to collapse (72). IL-15RA is expressed in multiple types of immune cells including dendritic cells (DC), macrophages, and natural killer cells (NK), and it plays a prominent role in TME (73). Disabled-2 (DAB2) is considered to be an immune-regulatory factor (74), and has recently been found to be involved in the regulation of tumor-related signaling pathways (75, 76). To sum up, TIPRGPI is likely to be a prognostic indicator strongly related to the immune status in HCC.

Systematically exploring the hallmark gene sets between the low- and high-risk groups provided us more insights into the transcriptomic regulatory mechanisms of TIPRGPI in HCC. Those hallmark pathways with an increased level in the high-risk group were found to be relevant to well-recognized oncogenic signaling pathways, including PI3K pathway, Wnt pathway, Myc pathway, and Cell cycle pathway (61). The similarities and disparities of mutation status or CNV profiles gave a hint of oncogenes that linked to the TIPRGPI model. These preliminary data strongly implied the inherent associations between immune-derived signature and oncogenic pathways and could provide more clues or new strategies for candidate drug discovery in future studies.

In consideration of the importance of immune infiltration in the tumor ecosystem, ssGSEA algorithm was used to estimate the activities of TME cells in low- and high-risk groups. 11 types of immune cells were significantly associated with the risk score. In line with the survival analysis, a higher abundance of activated B cell, effector memory CD8+ T cell, and activated CD8+ T cell in the low-risk group might contribute to a better prognosis. These results suggest that the poor survival outcomes of high-risk group patients are probably due to the low infiltration of these protective immune cells, which is also the main cause for the low objective response rates to immunotherapy, agreeing with previous observations (43, 77).

In the past decade, anti-cancer immunotherapies targeting PD-1/PD-L1 and CTLA-4, have achieved positive response in HCC patients (78, 79). However, as mentioned above, only a small proportion of HCC patients can respond to immunotherapies, and the main reason might be the limitations in their tumor immunity status (80). To determine whether TIPRGPI was capable of predicting the efficiency of anti-cancer immunotherapies in HCC patients, we measured the expression levels of 50 common immune checkpoints in low- and high-risk groups and found more than half of them were significantly associated with the risk level. It is widely known that cancer immunotherapy restores or enhances the anti-tumor function of CD8+ T cells in the tumor microenvironment (81). Thus, we estimated the expression levels of the key molecules in two CD8+ T cells anti-tumor related pathways (IFN-gamma (82) and m6A pathway (83)) in low- and high-risk groups, and the results indicated that the majority of these molecules were expressed differently between two groups of HCC patients. IPS, developed from a panel of immune-related genes belonging to the four classes-effector cells, immunosuppressive cells, MHC molecules, and selected immunomodulators, was a superior predictor of response to immune checkpoint inhibitors (50). Our study showed that the low-risk group had higher IPS, IPS-PD-11/PD-L1/PD-L2, and IPS-CTLA4 scores, suggesting that HCC patients in the low-risk group might have a better response to anti-CTLA-4 and anti-PD-1 antibodies. Also, TIDE analysis showed that the low-risk group had a lower TIDE score than the high-risk group in three different datasets. Taken together, these results suggested that patients in the low-risk group might have a better response to immunotherapies and TIPGPI could be a potential biomarker for predicting the efficiency of immunotherapies in HCC.

As another application of the prognostic classifier, we demonstrated the feasibility of searching candidate drugs by combining the core target and structure-based approaches. A PPI network was constructed for these signature genes, among which CD44 was found to be the hub node. Interestingly, CD44 was a well-defined cancer stem cell (CSC) marker that was involved in tumor initiation, epithelial-mesenchymal transition (EMT), and therapy resistance in multiple types of cancer. Therefore, substantial efforts have been exerted to develop effective anti-cancer drugs or antibodies by targeting CD44 (84–87). Preclinical and clinical trials of CD44 monoclonal antibodies have also been performed to evaluate the pharmacokinetics, efficacy, and drug-related toxicity in cancer (88). In the present study, we identified six drugs with high affinity to CD44 from a total of 9800 small molecules. Among them, Pentostatin has been reported in clinical trials for chronic lymphocytic leukemia (CLL) (89). Noticeably, Deoxynojirimycin could exert anti-HCV activity through inhibiting alpha-glucosidase (90). Although more in-depth investigations should be conducted for the specific mechanisms of the small compounds, our results indicated their potential in cancer immunotherapy especially for the immunological high-risk group of HCC patients.

## Conclusion

In summary, we constructed an immune-related TIPRGPI model to predict the prognosis and immunotherapy efficacy of HCC patients using a novel “TIP genes”- guided strategy, and it was well-validated from multiple aspects. Combining these results and the linkage between TIPRGPI and oncogenic hallmark pathways, our study provides new perspectives for the identification of prognostic classifiers and even the discovery of immunotherapeutic drugs. Notably, in the era, where immunotherapy offers new hope for effective cancer treatment, TIPRGPI provides certain guiding significance for clinical judgment and personalized treatment.

## Data Availability Statement

The data achieved and analyzed in the current study are available in the TCGA repository (https://portal.gdc.cancer.gov/), ICGC database (https://icgc.org/), and GEO database (https://www.ncbi.nlm.nih.gov/geo/) under the accession number GSE14520.

## Author Contributions

YT, YZ, and DW conceived the study. YT, YZ, and CG contributed to data collection, analysis, and interpretation. ZY helped with data visualization. YZ and CG completed the drafting of the manuscript. YT, YW, and DW revised the manuscript. DW supervised the research. All authors contributed to the article and approved the submitted version.

## Funding

This work was supported by the National Natural Science Foundation of China (Grant No. 82172723, 81673460), Science and Technology Department of Sichuan Province (Grant No. 2021ZYD0079), and the Sichuan Youth Science and Technology Innovation Research Team of Experimental Formulology (2020JDTD0022).

## Conflict of Interest

The authors declare that the research was conducted in the absence of any commercial or financial relationships that could be construed as a potential conflict of interest.

## Publisher’s Note

All claims expressed in this article are solely those of the authors and do not necessarily represent those of their affiliated organizations, or those of the publisher, the editors and the reviewers. Any product that may be evaluated in this article, or claim that may be made by its manufacturer, is not guaranteed or endorsed by the publisher.
