# Supplementary material for: Identification of a Tumor Immunological Phenotype-Related Gene Signature for Predicting Prognosis, Immunotherapy Efficacy, and Drug Candidates in Hepatocellular Carcinoma
Source: Front Immunol. 2022 Apr 12;13:862527. doi: 10.3389/fimmu.2022.862527 (PMC9039265; doi:10.3389/fimmu.2022.862527)
Supplement: Supplementary file 1 [file DataSheet_1.docx]

Supplementary Material

# Supplementary Table 1 Demographic information of HCC patients in the present study.

| **Variables** | **TCGA-LIHC**  **(Training)** | **ICGC-LIRI-JP**  **(Validation1)** | **GSE14520**  **(Validation2)** |
| --- | --- | --- | --- |
| **Total** | 336 | 238 | 238 |
| **Gender** |  |  |  |
| Female | 108 | 61 | 30 |
| Male | 228 | 177 | 191 |
| **Age** |  |  |  |
| <60 | 153 | 49 | 178 |
| ≥60 | 183 | 189 | 43 |
| **Race** |  |  |  |
| White | 163 | - | - |
| Others | 163 | - | - |
| **BMI** |  |  |  |
| <25 | 159 | - | - |
| ≥25 | 150 | - | - |
| **TNM stage** |  |  |  |
| I | 157 | 36 | 93 |
| II | 77 | 109 | 77 |
| III | 79 | 72 | 49 |
| VI | 4 | 21 | 0 |
| **Grade** |  |  |  |
| G1 | 52 | - | - |
| G2 | 157 | - | - |
| G3 | 110 | - | - |
| G4 | 12 | - | - |
| NA | 5 | - | - |
| **Progression** |  |  |  |
| Yes | 161 | 197 | 121 |
| No | 175 | 41 | 100 |
| **Tumor burden** |  |  |  |
| Tumor free | 207 | - | - |
| With tumor | 108 | - | - |

# Supplementary Table 2 Univeriate analysis of the black module.

| **Genes** | **HR** | **P** | **lower** | **upper** |
| --- | --- | --- | --- | --- |
| SLC1A5 | 1.360075677 | 1.35E-08 | 1.223124314 | 1.512361275 |
| SLC16A3 | 1.333078235 | 4.25E-07 | 1.192526801 | 1.490195087 |
| IMPDH1 | 1.389794003 | 1.02E-06 | 1.2179144 | 1.585930318 |
| S100A9 | 1.236257957 | 1.18E-06 | 1.134882504 | 1.346688957 |
| ST6GALNAC4 | 1.513088572 | 1.60E-06 | 1.277574048 | 1.792019047 |
| SMOX | 1.382002854 | 5.21E-06 | 1.202444016 | 1.588374896 |
| IL15RA | 1.476043684 | 9.47E-06 | 1.242415709 | 1.75360384 |
| DAB2 | 1.368444724 | 9.55E-06 | 1.191014749 | 1.572307115 |
| ADA | 1.577477527 | 9.75E-06 | 1.288945834 | 1.9305973 |
| PLBD1 | 1.279292136 | 1.62E-05 | 1.143806054 | 1.430826813 |
| MSC | 1.174130939 | 3.46E-05 | 1.088220269 | 1.266823916 |
| UAP1L1 | 1.282436449 | 4.54E-05 | 1.137931804 | 1.445291571 |
| RAB42 | 1.384436333 | 5.55E-05 | 1.18192188 | 1.621650292 |
| ANXA5 | 1.390231319 | 6.50E-05 | 1.182687172 | 1.634196401 |
| ABCC1 | 1.278488835 | 0.000195565 | 1.123445204 | 1.454929618 |
| MARCKS | 1.381715985 | 0.000206314 | 1.164822558 | 1.638995614 |
| RAB3IL1 | 1.302248964 | 0.000209143 | 1.132568888 | 1.497350299 |
| LHFPL2 | 1.365804283 | 0.00022127 | 1.157557178 | 1.611515504 |
| CTSC | 1.290119988 | 0.000275331 | 1.124655933 | 1.479927803 |
| CAPG | 1.222867858 | 0.000339584 | 1.095424066 | 1.365138712 |
| LPCAT4 | 1.334595899 | 0.000404449 | 1.137346263 | 1.566054483 |
| CD97 | 1.284834356 | 0.000446885 | 1.117069213 | 1.477795022 |
| CSF1 | 1.318772503 | 0.000499704 | 1.128514216 | 1.541106785 |
| IER3 | 1.211640102 | 0.000514066 | 1.08724295 | 1.350270182 |
| RGS2 | 1.190109265 | 0.000613237 | 1.077310341 | 1.314718711 |
| S100A11 | 1.209735799 | 0.000654071 | 1.084267923 | 1.349722402 |
| CXCL8 | 1.142283555 | 0.000679826 | 1.057903201 | 1.233394246 |
| CMTM7 | 1.372371874 | 0.000859062 | 1.139285244 | 1.653145751 |
| FABP5 | 1.297896986 | 0.000925506 | 1.112326441 | 1.514426454 |
| TREM2 | 1.215716258 | 0.000949795 | 1.082739635 | 1.365024399 |
| IKBKE | 1.285069927 | 0.001007991 | 1.106627872 | 1.49228549 |
| PTGS1 | 1.288040875 | 0.00108624 | 1.106581645 | 1.499256114 |
| MLXIPL | 0.796531619 | 0.001195704 | 0.69412271 | 0.914049649 |
| NCF2 | 1.240301317 | 0.001300681 | 1.087739293 | 1.414261088 |
| ENO2 | 1.203398871 | 0.001390054 | 1.07425878 | 1.348063307 |
| PLAUR | 1.260535891 | 0.001393099 | 1.093681038 | 1.452846559 |
| NFKBIE | 1.324264047 | 0.001598608 | 1.112317632 | 1.576595762 |
| TCN2 | 1.339403852 | 0.001649898 | 1.116526367 | 1.606771439 |
| SLC7A8 | 1.27687464 | 0.001661111 | 1.096467504 | 1.486965039 |
| IL4I1 | 1.210241778 | 0.00166728 | 1.074503178 | 1.363127807 |
| CD5L | 0.859909108 | 0.001958408 | 0.781561466 | 0.94611071 |
| HMOX1 | 1.234940512 | 0.002011757 | 1.080157882 | 1.411902921 |
| SH3BGRL3 | 1.331041675 | 0.002102609 | 1.109283025 | 1.597132472 |
| PAG1 | 1.28143736 | 0.002682402 | 1.089889618 | 1.506649554 |
| ADAP2 | 1.374275752 | 0.002988913 | 1.11409338 | 1.695220415 |
| PLA2G7 | 1.212681163 | 0.003244378 | 1.066558077 | 1.378823746 |
| CXCL1 | 1.108533313 | 0.003628851 | 1.03418022 | 1.188232072 |
| KLRB1 | 0.788333622 | 0.003717909 | 0.671319627 | 0.925743676 |
| RGS19 | 1.34730685 | 0.003777073 | 1.101163783 | 1.648470261 |
| ARPC1B | 1.306043898 | 0.004567946 | 1.085974849 | 1.57070918 |
| FCER1G | 1.232617566 | 0.004721255 | 1.066160555 | 1.425063097 |
| CHST11 | 1.214462909 | 0.005314425 | 1.059370499 | 1.392260931 |
| EVA1B | 1.288182918 | 0.005808679 | 1.076052369 | 1.542132408 |
| SLC7A7 | 1.239975569 | 0.005989352 | 1.063644825 | 1.445538375 |
| SDC3 | 1.2920466 | 0.006070575 | 1.075953806 | 1.551539114 |
| RASA3 | 1.238303626 | 0.006184279 | 1.062610874 | 1.443045529 |
| PLTP | 1.153233315 | 0.006647668 | 1.04040524 | 1.278297175 |
| TMSB10 | 1.169640752 | 0.007005456 | 1.043733844 | 1.310735966 |
| SLC2A6 | 1.193743057 | 0.007668595 | 1.048033422 | 1.359710918 |
| MMP9 | 1.125846386 | 0.007763733 | 1.031760362 | 1.228512096 |
| PLEKHA4 | 1.227610197 | 0.008776676 | 1.053058248 | 1.431095381 |
| TGFB1 | 1.183036754 | 0.008824713 | 1.043191999 | 1.34162835 |
| GZMH | 0.792096028 | 0.009110523 | 0.664819614 | 0.94373888 |
| ELF4 | 1.201134385 | 0.00988366 | 1.045020482 | 1.380569889 |
| SLC43A2 | 1.244606583 | 0.01016434 | 1.053326189 | 1.470622836 |
| S100A6 | 1.130609436 | 0.010365157 | 1.029319221 | 1.241867121 |
| ARHGAP4 | 1.20037785 | 0.010904074 | 1.042921299 | 1.381606631 |
| FABP3 | 1.129223974 | 0.010950974 | 1.028302937 | 1.240049733 |
| TNFRSF4 | 1.266999033 | 0.011153036 | 1.05536674 | 1.521069869 |
| LGALS1 | 1.192624755 | 0.011499913 | 1.040326845 | 1.367218209 |
| CARD6 | 1.247998797 | 0.011774171 | 1.050383468 | 1.482792756 |
| BATF | 1.15413244 | 0.012060455 | 1.031928826 | 1.290807715 |
| LIF | 1.15315659 | 0.012269118 | 1.031459608 | 1.289212016 |
| NFE2L3 | 1.199203842 | 0.012453074 | 1.039963825 | 1.382826807 |
| IFI27L2 | 1.169196399 | 0.012837641 | 1.033744394 | 1.322396744 |
| FCGR2A | 1.235726564 | 0.012957544 | 1.045730149 | 1.46024301 |
| TNFAIP8L3 | 1.209264419 | 0.013153577 | 1.040622238 | 1.405236579 |
| AKR1B1 | 1.166900106 | 0.013476972 | 1.032432173 | 1.318881659 |
| IL27RA | 1.24396936 | 0.0135789 | 1.045979184 | 1.479436486 |
| TLR2 | 1.190649947 | 0.014043946 | 1.035879946 | 1.368544011 |
| PPP1R18 | 1.226355945 | 0.014151167 | 1.041892578 | 1.443477893 |
| GAL3ST4 | 1.232755473 | 0.014334516 | 1.042653946 | 1.457517196 |
| RPP25 | 1.178337418 | 0.016689265 | 1.030169519 | 1.347816107 |
| ICAM1 | 1.160042144 | 0.016780491 | 1.027149425 | 1.310128538 |
| STK17A | 1.268564337 | 0.018479256 | 1.040786976 | 1.546191021 |
| GMIP | 1.255944828 | 0.018611194 | 1.038818474 | 1.518453368 |
| CCDC109B | 1.198404651 | 0.020051392 | 1.028850056 | 1.395901861 |
| ACSS1 | 1.175646751 | 0.021222199 | 1.024457693 | 1.34914823 |
| ITGAM | 1.185076902 | 0.021853191 | 1.024963719 | 1.370201927 |
| LGALS9 | 1.191172205 | 0.022755162 | 1.02469164 | 1.384700691 |
| SIGLEC9 | 1.29512653 | 0.023154138 | 1.036041671 | 1.61900122 |
| MSN | 1.22227845 | 0.023217499 | 1.027780229 | 1.453583722 |
| GPR34 | 1.233998863 | 0.023433076 | 1.02882852 | 1.480084547 |
| MX2 | 1.229597966 | 0.024020509 | 1.027558477 | 1.471362644 |
| CPVL | 1.160534525 | 0.024271126 | 1.019541709 | 1.321025293 |
| PLEKHA2 | 1.233176229 | 0.024499455 | 1.027319571 | 1.480282918 |
| RAB7B | 1.239028606 | 0.025709502 | 1.02634274 | 1.495788714 |
| SH3BP1 | 1.208672164 | 0.025725785 | 1.023240026 | 1.427708419 |
| TNFAIP8 | 1.224038593 | 0.025749622 | 1.02477892 | 1.462042639 |
| FAM105A | 1.228746795 | 0.025880604 | 1.025091388 | 1.47286252 |
| ITGB7 | 0.756150561 | 0.027396495 | 0.589856945 | 0.969325995 |
| SLAMF6 | 0.80135034 | 0.029046653 | 0.656848847 | 0.977641006 |
| TM7SF2 | 0.859413001 | 0.029699844 | 0.749693516 | 0.9851902 |
| CCR7 | 0.800847328 | 0.029905335 | 0.655373381 | 0.978612286 |
| EMILIN2 | 1.206407255 | 0.030952386 | 1.017343774 | 1.430606352 |
| DUSP5 | 1.161835766 | 0.031881449 | 1.013081611 | 1.332431989 |
| SEL1L3 | 1.116664581 | 0.032036221 | 1.009514886 | 1.235187122 |
| CMTM3 | 1.180655037 | 0.032383213 | 1.014043939 | 1.374640943 |
| DOK1 | 1.240547288 | 0.033670449 | 1.016786066 | 1.513551006 |
| RPS6KA1 | 1.22143438 | 0.033918276 | 1.015306494 | 1.469410422 |
| RGS1 | 1.117822086 | 0.035975138 | 1.007315643 | 1.240451516 |
| CD7 | 1.132725664 | 0.035992891 | 1.008177875 | 1.272659776 |
| OSMR | 1.144631978 | 0.03739582 | 1.007913357 | 1.299895825 |
| STAB1 | 1.216259485 | 0.037535933 | 1.011352296 | 1.462682332 |
| SLFN11 | 1.205251607 | 0.038298678 | 1.010114952 | 1.438085272 |
| GZMA | 0.860764776 | 0.038569051 | 0.746780995 | 0.992146298 |
| BLVRA | 1.162757195 | 0.03903886 | 1.007614155 | 1.341787715 |
| MICAL1 | 1.18083611 | 0.039218067 | 1.008250906 | 1.382963219 |
| RAB20 | 1.203819873 | 0.040594004 | 1.007975514 | 1.437715765 |
| APOL4 | 1.201142244 | 0.04195931 | 1.006686607 | 1.433159714 |
| PLAU | 1.171069123 | 0.041969205 | 1.005751471 | 1.363560413 |
| GZMK | 0.855888426 | 0.043374371 | 0.735945655 | 0.995379201 |
| PRNP | 1.160672418 | 0.044063694 | 1.003968408 | 1.34183551 |
| IL7R | 0.871005529 | 0.044560909 | 0.761200867 | 0.99664972 |
| SELM | 1.104435986 | 0.046760686 | 1.001424618 | 1.218043599 |
| SLC9A9 | 1.215731189 | 0.04912166 | 1.000751885 | 1.476891871 |
| PTGER4 | 1.200740579 | 0.049781596 | 1.000174566 | 1.441526296 |
| CST7 | 0.859043775 | 0.04991359 | 0.737998512 | 0.999942676 |
| TRPV2 | 1.168279123 | 0.050630879 | 0.999572877 | 1.365459329 |
| GPR171 | 0.809687384 | 0.051222612 | 0.654859061 | 1.001121767 |
| CD209 | 1.20197892 | 0.051705349 | 0.998640701 | 1.446719849 |
| GIMAP7 | 0.851648866 | 0.051944977 | 0.724325205 | 1.001353792 |
| TMSB4X | 1.175885443 | 0.055626184 | 0.996112603 | 1.388102682 |
| TMEM156 | 1.106496123 | 0.05616884 | 0.997341234 | 1.227597563 |
| CD8A | 0.872928488 | 0.058771505 | 0.758174552 | 1.00505107 |
| CD69 | 0.839874646 | 0.058911622 | 0.70077042 | 1.006591318 |
| MTHFD2 | 1.170784223 | 0.058982984 | 0.994035633 | 1.378960323 |
| RGL1 | 1.20079451 | 0.059522648 | 0.992680542 | 1.452539256 |
| LY96 | 1.111292375 | 0.060347375 | 0.995419504 | 1.240653552 |
| LAIR1 | 1.177849378 | 0.061018018 | 0.992461729 | 1.397866656 |
| CD79A | 0.890502974 | 0.062511277 | 0.788205576 | 1.006077057 |
| RGS10 | 1.157661301 | 0.062957375 | 0.99212086 | 1.350823011 |
| RELB | 1.193828719 | 0.063165211 | 0.990327225 | 1.439147561 |
| NKG7 | 0.876429612 | 0.06444619 | 0.76207727 | 1.00794092 |
| MSR1 | 1.186581513 | 0.068501242 | 0.987099038 | 1.426377326 |
| CD83 | 1.199385004 | 0.069096702 | 0.98588056 | 1.459126438 |
| CD44 | 1.115973197 | 0.070075252 | 0.991042559 | 1.256652568 |
| RNASE1 | 1.154117406 | 0.070842743 | 0.98789596 | 1.348306948 |
| CD300A | 1.178261367 | 0.071530743 | 0.98573219 | 1.408394555 |
| S1PR4 | 0.829024478 | 0.071553404 | 0.676073313 | 1.016578487 |
| EMP3 | 1.133714589 | 0.071908787 | 0.988886075 | 1.299754139 |
| LAT2 | 1.184833962 | 0.072621635 | 0.984554477 | 1.425854588 |
| PYCARD | 1.110617292 | 0.077261651 | 0.988595117 | 1.24770065 |
| CXCR6 | 0.839278558 | 0.07788089 | 0.690743099 | 1.019754666 |
| GNA15 | 1.186952218 | 0.078751379 | 0.980500517 | 1.436873867 |
| HAVCR2 | 1.150889803 | 0.079600153 | 0.983545849 | 1.346706248 |
| CYBB | 1.126095564 | 0.079829304 | 0.985978271 | 1.286124915 |
| GIMAP6 | 0.839698495 | 0.080198791 | 0.6904313 | 1.021236381 |
| PRF1 | 0.857639728 | 0.080374329 | 0.72201204 | 1.018744651 |
| IGJ | 0.931299593 | 0.080951816 | 0.859755127 | 1.008797626 |
| FCGR2B | 1.143698725 | 0.083161582 | 0.98253731 | 1.331294762 |
| VSIG4 | 1.129732323 | 0.088392072 | 0.981838931 | 1.299902744 |
| TMC6 | 1.121920792 | 0.088395662 | 0.982861599 | 1.280654635 |
| APBB1IP | 1.11906608 | 0.090761105 | 0.982296209 | 1.274879083 |
| ITM2A | 0.886057058 | 0.091790474 | 0.769816558 | 1.019849602 |
| F13A1 | 1.102472706 | 0.093292169 | 0.983759606 | 1.235511257 |
| CETP | 0.869268172 | 0.0945363 | 0.737607948 | 1.024429248 |
| HK3 | 1.157358307 | 0.095320419 | 0.974741906 | 1.374187609 |
| MZB1 | 0.911073865 | 0.099874473 | 0.815410971 | 1.017959799 |
| CCDC88B | 1.172171664 | 0.100399578 | 0.969809278 | 1.416759398 |
| SIGLEC10 | 1.157285444 | 0.101766349 | 0.97152425 | 1.378565278 |
| MILR1 | 1.193484056 | 0.102008551 | 0.965479849 | 1.475332907 |
| FCHSD1 | 1.187766854 | 0.102389752 | 0.966179457 | 1.460173975 |
| MMP19 | 1.153802492 | 0.10435759 | 0.970846417 | 1.371236653 |
| LILRB4 | 1.146408184 | 0.106762413 | 0.971028897 | 1.353463042 |
| CD6 | 0.855420353 | 0.107409498 | 0.707317447 | 1.034534045 |
| YBX3 | 1.120648796 | 0.109731402 | 0.974648 | 1.288520289 |
| MVP | 1.175684764 | 0.110202518 | 0.963917124 | 1.433976667 |
| SELL | 0.870773338 | 0.116844955 | 0.732479261 | 1.035177711 |
| ALOX5 | 1.09831226 | 0.119097753 | 0.976138704 | 1.235777063 |
| IL18 | 1.117057708 | 0.119505184 | 0.971744228 | 1.284101193 |
| LRRC25 | 1.154936071 | 0.119914829 | 0.96319645 | 1.384844523 |
| PLK3 | 1.164317824 | 0.126776819 | 0.957773049 | 1.415404199 |
| CYBA | 1.083478001 | 0.129529748 | 0.976790969 | 1.2018176 |
| CFP | 0.860896447 | 0.130024502 | 0.709155147 | 1.045106554 |
| GLIPR1 | 1.122008634 | 0.131041927 | 0.966277607 | 1.302838196 |
| CCL5 | 0.910984619 | 0.131249035 | 0.807102638 | 1.028237224 |
| GZMB | 0.874521651 | 0.131683457 | 0.734621876 | 1.041063631 |
| PLEKHO1 | 1.142725882 | 0.132619763 | 0.960342967 | 1.35974593 |
| CD5 | 0.875771839 | 0.133862444 | 0.736323258 | 1.04162989 |
| ATP2A3 | 0.880360843 | 0.135488954 | 0.744737814 | 1.040681969 |
| PARP8 | 1.157793355 | 0.136264171 | 0.95481991 | 1.403914433 |
| LYN | 1.18143876 | 0.141675216 | 0.945882496 | 1.475656384 |
| ALOX5AP | 1.101155265 | 0.146894231 | 0.966729284 | 1.254273495 |
| PTAFR | 1.123797763 | 0.146907062 | 0.95983962 | 1.315762951 |
| CD2 | 0.909660273 | 0.146972742 | 0.800401883 | 1.033832916 |
| CD3E | 0.905806857 | 0.149879575 | 0.791690829 | 1.036371816 |
| PIK3IP1 | 0.886009615 | 0.149933103 | 0.751425764 | 1.04469806 |
| MAP4K1 | 0.863950443 | 0.151729085 | 0.707374999 | 1.055183416 |
| NBL1 | 1.097276633 | 0.153746562 | 0.965873092 | 1.246557149 |
| LCK | 0.885345512 | 0.153771528 | 0.748926374 | 1.046613796 |
| SERPINB9 | 1.123890227 | 0.158174351 | 0.955602438 | 1.321814587 |
| LDHB | 1.104779756 | 0.160734997 | 0.961176853 | 1.269837393 |
| AMIGO2 | 1.109813674 | 0.160978025 | 0.959360911 | 1.283861347 |
| STX11 | 1.120761814 | 0.163133888 | 0.954834214 | 1.315523707 |
| HMHA1 | 1.156373961 | 0.168662587 | 0.940275481 | 1.422137197 |
| TNFSF13B | 1.127654724 | 0.170642807 | 0.949596379 | 1.3391007 |
| SLAMF8 | 1.103090448 | 0.173635811 | 0.957700689 | 1.270552011 |
| HLA.B | 0.906795528 | 0.174842013 | 0.787281356 | 1.044452689 |
| SPI1 | 1.117486791 | 0.182113108 | 0.949243273 | 1.315549725 |
| SCN1B | 1.136870827 | 0.183673336 | 0.940985599 | 1.373533536 |
| CYTH4 | 1.145575136 | 0.185678607 | 0.936729079 | 1.400983937 |
| B2M | 0.8713856 | 0.185722379 | 0.710652909 | 1.068472181 |
| C5AR1 | 1.124436439 | 0.185992221 | 0.94504017 | 1.337887368 |
| HLA.DQB2 | 1.078099225 | 0.188280514 | 0.963845081 | 1.205897048 |
| TBC1D10C | 0.888444088 | 0.194281081 | 0.743127404 | 1.062177082 |
| HLA.DRB5 | 0.929690791 | 0.194365905 | 0.832763491 | 1.037899688 |
| TNFSF12 | 1.130273578 | 0.198427227 | 0.937843842 | 1.362186649 |
| ANKRD22 | 1.071456579 | 0.203588448 | 0.963310242 | 1.191743999 |
| STK17B | 1.112121931 | 0.204120974 | 0.94388842 | 1.310340462 |
| SLC2A3 | 1.10060648 | 0.205395478 | 0.948846382 | 1.276639345 |
| CD8B | 0.903999592 | 0.206770387 | 0.772896845 | 1.057340662 |
| ZNF385A | 1.095785256 | 0.20988058 | 0.949796401 | 1.264213388 |
| FCGR3A | 1.090322889 | 0.211027435 | 0.952148868 | 1.248548458 |
| CCL19 | 0.953978954 | 0.211086036 | 0.886076486 | 1.027084974 |
| CD96 | 0.874377603 | 0.214149196 | 0.707478906 | 1.080648746 |
| TMC8 | 0.888547595 | 0.214552047 | 0.73729185 | 1.070833521 |
| STAT4 | 0.891150389 | 0.215210553 | 0.742680773 | 1.069300626 |
| SYK | 1.108208055 | 0.216411053 | 0.941610342 | 1.304281653 |
| IGLL5 | 0.944243557 | 0.217905939 | 0.861885217 | 1.034471735 |
| PLEK | 1.089240777 | 0.219132844 | 0.950412704 | 1.248347655 |
| SIT1 | 0.897838423 | 0.219414139 | 0.755970621 | 1.06632958 |
| C3AR1 | 1.115457091 | 0.220192712 | 0.936682879 | 1.328351943 |
| TNFRSF18 | 1.093436252 | 0.221473697 | 0.947554449 | 1.26177745 |
| DOCK10 | 1.134675467 | 0.223033479 | 0.925998734 | 1.390378159 |
| FGR | 1.118312488 | 0.223814755 | 0.93393617 | 1.339088112 |
| ARL4C | 1.086533958 | 0.224129241 | 0.950451926 | 1.24209969 |
| PCED1B | 1.106542002 | 0.225842359 | 0.939324001 | 1.30352807 |
| CXCR3 | 0.901590799 | 0.228610357 | 0.761670526 | 1.067214682 |
| CASP1 | 1.123461045 | 0.230997359 | 0.928601396 | 1.359210449 |
| CD48 | 0.906684525 | 0.232496992 | 0.771999917 | 1.064866471 |
| FERMT3 | 1.107933514 | 0.235228284 | 0.9354336 | 1.31224351 |
| CD27 | 0.918844687 | 0.236109419 | 0.798791488 | 1.056941107 |
| HCK | 1.103363109 | 0.237643316 | 0.937168374 | 1.29903034 |
| BATF2 | 1.120198501 | 0.237760293 | 0.927808899 | 1.352481835 |
| EBI3 | 1.11527248 | 0.237902701 | 0.930460974 | 1.336791911 |
| FXYD5 | 1.095615189 | 0.238648547 | 0.941229511 | 1.27532406 |
| F10 | 0.919128088 | 0.239917023 | 0.798537602 | 1.057929445 |
| SAMD9L | 1.113664755 | 0.242372883 | 0.929758797 | 1.333947246 |
| CSF2RA | 1.105120532 | 0.243471135 | 0.934250337 | 1.30724212 |
| CXCL9 | 0.944904846 | 0.244826638 | 0.858836905 | 1.039598046 |
| CLEC7A | 1.118029413 | 0.248130765 | 0.925175377 | 1.35108413 |
| RASAL3 | 0.881784365 | 0.248162593 | 0.712248575 | 1.091674584 |
| GLIPR2 | 1.092412051 | 0.249113955 | 0.939952523 | 1.26960039 |
| PALLD | 1.095497627 | 0.255784911 | 0.936038441 | 1.282121543 |
| PLEKHO2 | 1.122598745 | 0.259695406 | 0.918095246 | 1.372654904 |
| TYROBP | 1.097303385 | 0.260805461 | 0.933335969 | 1.290076414 |
| NCF4 | 1.10245947 | 0.261522727 | 0.929852826 | 1.307106725 |
| VCAM1 | 1.063153663 | 0.262710258 | 0.955111704 | 1.183417296 |
| CCL18 | 0.934921844 | 0.263267746 | 0.830947617 | 1.051906085 |
| PSTPIP1 | 0.892819175 | 0.269106901 | 0.730198976 | 1.091655982 |
| IL2RG | 1.068092673 | 0.271893916 | 0.949673176 | 1.20127849 |
| ANO9 | 1.081387029 | 0.274345281 | 0.939836744 | 1.244256424 |
| GPNMB | 1.067030552 | 0.274559152 | 0.949801389 | 1.198728716 |
| LAPTM5 | 1.088247363 | 0.274899753 | 0.934971354 | 1.266650917 |
| SAMSN1 | 1.099423014 | 0.27651294 | 0.926876033 | 1.304091293 |
| CLIC2 | 1.106451006 | 0.276906658 | 0.922018743 | 1.327775425 |
| RENBP | 1.062097272 | 0.281271132 | 0.951853799 | 1.185109117 |
| CD86 | 1.104207846 | 0.281645704 | 0.921888536 | 1.322583935 |
| UBE2L6 | 1.113553218 | 0.285792617 | 0.913986661 | 1.356694601 |
| IL18R1 | 0.906343881 | 0.28780349 | 0.756042398 | 1.086525349 |
| FGD3 | 1.109415584 | 0.288475679 | 0.915860945 | 1.343875338 |
| ITGB2 | 1.083729928 | 0.292109452 | 0.933153657 | 1.258603606 |
| LAMP3 | 1.087363664 | 0.293485123 | 0.930056151 | 1.2712778 |
| HLA.F | 0.926489875 | 0.296591894 | 0.802740103 | 1.069316813 |
| MPEG1 | 0.910649779 | 0.297644006 | 0.763582647 | 1.086042255 |
| CCR1 | 1.09276016 | 0.304826111 | 0.922445958 | 1.294520027 |
| CSF1R | 1.088880795 | 0.306589234 | 0.924886715 | 1.281953094 |
| PILRA | 1.114724105 | 0.307094091 | 0.905008077 | 1.373037283 |
| TNFAIP8L2 | 1.094146371 | 0.317462131 | 0.917202017 | 1.305226395 |
| TAP1 | 1.082875747 | 0.327733248 | 0.923272853 | 1.270068627 |
| AXL | 1.092073008 | 0.331232196 | 0.914305026 | 1.304404351 |
| UBD | 1.045323629 | 0.332459859 | 0.955695968 | 1.143356807 |
| CXCR4 | 1.068428301 | 0.334831358 | 0.933958175 | 1.222259267 |
| LPAR6 | 1.113418757 | 0.339432425 | 0.893159806 | 1.38799498 |
| SLC1A3 | 1.086860727 | 0.339876516 | 0.915983385 | 1.289615357 |
| HLA.DRB1 | 0.939236932 | 0.343359247 | 0.825014813 | 1.069272939 |
| HLA.DPB1 | 0.933922635 | 0.34397799 | 0.810627772 | 1.075970401 |
| WIPF1 | 1.083407407 | 0.351071286 | 0.915516378 | 1.282086958 |
| GPR183 | 1.065865894 | 0.351789822 | 0.931944747 | 1.219031609 |
| WAS | 0.907206555 | 0.361286466 | 0.736045199 | 1.1181701 |
| AMICA1 | 1.085044392 | 0.361966433 | 0.910408871 | 1.29317867 |
| TMEM106A | 1.091637153 | 0.365454415 | 0.902846196 | 1.319905515 |
| NFAM1 | 1.098907307 | 0.365897991 | 0.895718115 | 1.348188956 |
| S100A4 | 1.057986973 | 0.374177937 | 0.934306124 | 1.19804035 |
| CXCL10 | 0.959085167 | 0.379766716 | 0.873719225 | 1.052791711 |
| HAPLN3 | 1.075636161 | 0.382327426 | 0.913321844 | 1.266796757 |
| CFD | 1.058152866 | 0.394873051 | 0.928961708 | 1.205310701 |
| CTSW | 0.937930704 | 0.395686541 | 0.809006215 | 1.0874008 |
| TRIM22 | 0.943392811 | 0.400278045 | 0.823610589 | 1.080595621 |
| CXCL13 | 1.036252047 | 0.403647184 | 0.953168086 | 1.126578115 |
| RNASE6 | 1.068980557 | 0.404199347 | 0.91390355 | 1.250372023 |
| CD1C | 0.93414625 | 0.407613326 | 0.795046037 | 1.097583253 |
| SAMD9 | 1.086947106 | 0.422750237 | 0.886508725 | 1.332704324 |
| CCR5 | 0.928911099 | 0.426828618 | 0.774429801 | 1.114207936 |
| FPR1 | 1.062583118 | 0.431691866 | 0.913376026 | 1.236164351 |
| SH3BGRL | 1.063251318 | 0.434680362 | 0.911610495 | 1.240116664 |
| SOD2 | 1.062053107 | 0.439585634 | 0.911680216 | 1.237228561 |
| GIMAP2 | 1.074849144 | 0.440618522 | 0.894693358 | 1.291281165 |
| GSTP1 | 1.046922998 | 0.441595972 | 0.931516885 | 1.176626835 |
| HLA.DRA | 0.951156448 | 0.446639444 | 0.836068381 | 1.082086836 |
| CD52 | 0.952377279 | 0.449068048 | 0.839345657 | 1.080630459 |
| MS4A7 | 0.933606602 | 0.4496944 | 0.781276578 | 1.115637293 |
| CSF2RB | 1.073286079 | 0.452360836 | 0.892494927 | 1.290699782 |
| C1QB | 1.053567352 | 0.453533578 | 0.919184428 | 1.20759679 |
| MNDA | 1.062520479 | 0.454374506 | 0.906440864 | 1.245475368 |
| CD74 | 0.948602957 | 0.457594178 | 0.825316175 | 1.090306477 |
| TNFRSF1B | 0.925353515 | 0.459547788 | 0.753391875 | 1.136565387 |
| FPR3 | 1.058759846 | 0.462923622 | 0.909045748 | 1.233130912 |
| HLA.DMA | 0.951325477 | 0.464569539 | 0.832247026 | 1.087441752 |
| PDE4B | 1.071835382 | 0.466421457 | 0.889304422 | 1.291831074 |
| LFNG | 1.072525534 | 0.469250845 | 0.887270075 | 1.296460969 |
| IFITM1 | 0.956634927 | 0.480889786 | 0.845686322 | 1.082139275 |
| ITGAL | 0.941416541 | 0.480950444 | 0.795920368 | 1.113509767 |
| C1QC | 1.053365245 | 0.481223509 | 0.91148219 | 1.217334086 |
| COTL1 | 1.055553148 | 0.483542689 | 0.907391741 | 1.227906756 |
| RUNX3 | 0.936783521 | 0.486120789 | 0.779526027 | 1.125765316 |
| IL10RA | 0.937480557 | 0.486214072 | 0.781710203 | 1.124291063 |
| BIN2 | 0.929705395 | 0.488792451 | 0.756343719 | 1.142803331 |
| BCL2A1 | 1.04821743 | 0.490708792 | 0.91683191 | 1.198430998 |
| CD4 | 0.947101768 | 0.495347131 | 0.810115968 | 1.107251054 |
| IL18BP | 1.066398006 | 0.505615867 | 0.882503261 | 1.288612471 |
| PPP1R16B | 0.931085275 | 0.510666026 | 0.752647084 | 1.15182774 |
| C10orf54 | 1.066498261 | 0.512748942 | 0.8795061 | 1.293246903 |
| BASP1 | 1.036797804 | 0.515246601 | 0.929868356 | 1.156023517 |
| HLA.DQA2 | 1.030821755 | 0.517517564 | 0.940280298 | 1.130081628 |
| MARCO | 1.035336003 | 0.520607105 | 0.93125558 | 1.151048824 |
| CIITA | 0.93319384 | 0.529258461 | 0.752358396 | 1.157494551 |
| NLRC5 | 0.939436237 | 0.531403677 | 0.772499683 | 1.142447645 |
| AKNA | 0.938758378 | 0.533961986 | 0.769245254 | 1.145625909 |
| CD300LF | 1.066390091 | 0.534073557 | 0.870807682 | 1.305900086 |
| PSMB9 | 1.050612353 | 0.53656663 | 0.898338179 | 1.22869799 |
| PTPRC | 0.956241239 | 0.540451305 | 0.828603941 | 1.103539654 |
| SIGLEC1 | 1.063618028 | 0.542220598 | 0.872258687 | 1.29695849 |
| RCSD1 | 1.063886399 | 0.543394204 | 0.871265603 | 1.299092109 |
| GYPC | 1.054484606 | 0.549097982 | 0.886472598 | 1.254339714 |
| SASH3 | 0.951359787 | 0.549605648 | 0.807998971 | 1.12015668 |
| CD37 | 0.950113154 | 0.550883471 | 0.803049578 | 1.124108685 |
| RASSF5 | 0.946214913 | 0.551366031 | 0.788848547 | 1.13497409 |
| SIGLEC14 | 1.058826291 | 0.552786457 | 0.87671093 | 1.278771685 |
| PTPN7 | 0.944473485 | 0.557581809 | 0.780315307 | 1.143166302 |
| PSMB10 | 1.06511408 | 0.562245519 | 0.86047734 | 1.318417058 |
| BIRC3 | 1.033371984 | 0.565109333 | 0.924024769 | 1.155659125 |
| FGL2 | 0.954523724 | 0.567333323 | 0.813811283 | 1.119566119 |
| CD163L1 | 1.060124019 | 0.570643189 | 0.866406564 | 1.297154226 |
| HLA.DQB1 | 0.966344037 | 0.574479683 | 0.857491507 | 1.089014631 |
| ABI3 | 1.064004619 | 0.576043447 | 0.856058678 | 1.322462886 |
| ARHGEF6 | 0.944102051 | 0.576678521 | 0.771456861 | 1.1553837 |
| CLEC10A | 0.952909198 | 0.581011964 | 0.802893764 | 1.130954031 |
| TAP2 | 1.062724233 | 0.590444955 | 0.851536259 | 1.326288556 |
| ADAMDEC1 | 1.037888682 | 0.605624929 | 0.901245347 | 1.195249352 |
| GBP4 | 0.959093352 | 0.608987766 | 0.817255835 | 1.12554725 |
| XCL2 | 0.957595353 | 0.610508784 | 0.810532905 | 1.131340697 |
| DENND2D | 1.044461184 | 0.612767842 | 0.88253566 | 1.236096413 |
| FAIM3 | 0.956794803 | 0.616096102 | 0.805078972 | 1.137101238 |
| GBP1 | 0.963594735 | 0.621267054 | 0.831767737 | 1.116315015 |
| CCL3L3 | 1.043456772 | 0.629034804 | 0.87805218 | 1.240019738 |
| AIF1 | 1.040799304 | 0.631655291 | 0.883820502 | 1.225659721 |
| GBP5 | 0.965493556 | 0.632142345 | 0.836199353 | 1.114779392 |
| MS4A4A | 1.044014083 | 0.632724137 | 0.874957995 | 1.245734551 |
| CTSS | 1.036921385 | 0.634477255 | 0.892964389 | 1.204086044 |
| NCKAP1L | 1.046571057 | 0.63474736 | 0.867376756 | 1.262785716 |
| IGSF6 | 1.044424856 | 0.636994956 | 0.871915519 | 1.251065335 |
| DOK2 | 1.043399657 | 0.637355146 | 0.874454675 | 1.244984874 |
| SLAMF7 | 0.967349194 | 0.644998423 | 0.839948794 | 1.114073227 |
| ITGAX | 1.042510049 | 0.645161383 | 0.873226263 | 1.244611217 |
| APOBEC3C | 1.03333674 | 0.645273487 | 0.898679453 | 1.188170949 |
| INPP5D | 0.954210041 | 0.647526486 | 0.780513258 | 1.16656161 |
| SLA | 0.958895248 | 0.652758532 | 0.798664091 | 1.151272615 |
| TLR4 | 1.044177027 | 0.661730084 | 0.860344753 | 1.267289258 |
| PIK3CD | 1.042811519 | 0.664105631 | 0.863048426 | 1.260017204 |
| LTB | 0.976902098 | 0.669800393 | 0.8774116 | 1.087673914 |
| LCP2 | 1.04090257 | 0.671142218 | 0.865049795 | 1.252503804 |
| HLA.A | 0.962763917 | 0.678194018 | 0.804773576 | 1.151770371 |
| ARHGAP30 | 0.961465445 | 0.68175246 | 0.796827573 | 1.160120249 |
| HLA.DPA1 | 0.975313527 | 0.691337754 | 0.862095784 | 1.103399986 |
| CD3D | 1.023967227 | 0.694646932 | 0.909765891 | 1.152504059 |
| CARD11 | 1.036448321 | 0.699322834 | 0.864272018 | 1.242924798 |
| MS4A6A | 1.033658226 | 0.7010169 | 0.872943171 | 1.223962066 |
| IDO1 | 1.028199534 | 0.701852261 | 0.891751114 | 1.185526168 |
| FMNL1 | 1.033453746 | 0.706702814 | 0.870669954 | 1.22667222 |
| IFI16 | 1.028810295 | 0.70972282 | 0.885898408 | 1.194776528 |
| CCL3 | 1.03155319 | 0.714567824 | 0.87335144 | 1.218412125 |
| CD72 | 0.965801805 | 0.71488613 | 0.801322331 | 1.164042347 |
| UCP2 | 1.028764075 | 0.715570362 | 0.883224943 | 1.198285363 |
| CD163 | 1.028052956 | 0.720236391 | 0.883608386 | 1.196110061 |
| C1orf162 | 1.032140099 | 0.724617991 | 0.865574654 | 1.230758293 |
| CYTIP | 0.971580968 | 0.726689942 | 0.826549375 | 1.142060723 |
| GMFG | 0.965642953 | 0.727368741 | 0.793334433 | 1.175376077 |
| ARHGAP9 | 0.966647977 | 0.727761647 | 0.798589165 | 1.170073865 |
| FCN1 | 0.965049552 | 0.735480998 | 0.785083494 | 1.186269544 |
| IRF1 | 1.034603011 | 0.74598978 | 0.842140451 | 1.271050914 |
| EMB | 0.973104643 | 0.746587955 | 0.824789573 | 1.148089983 |
| FYB | 1.025727457 | 0.757238186 | 0.873135706 | 1.20498659 |
| LY86 | 1.027907044 | 0.758035594 | 0.862780862 | 1.224636447 |
| C1QA | 1.022286092 | 0.75836418 | 0.888351062 | 1.176414256 |
| IL2RB | 0.975537297 | 0.762251324 | 0.83091869 | 1.145326287 |
| RARRES3 | 0.977236866 | 0.762592775 | 0.841620021 | 1.134706719 |
| SELPLG | 0.97588211 | 0.764304829 | 0.831934927 | 1.144736038 |
| THEMIS2 | 1.024367375 | 0.779075329 | 0.865772309 | 1.212014415 |
| EVL | 1.025599131 | 0.781315788 | 0.857970847 | 1.225978227 |
| CECR1 | 0.97868041 | 0.789561626 | 0.835425452 | 1.146500077 |
| SLC15A3 | 1.027590214 | 0.794911271 | 0.836943574 | 1.261664084 |
| GIMAP4 | 0.97393318 | 0.796322326 | 0.796938783 | 1.190236766 |
| APOBEC3G | 0.975142115 | 0.799956782 | 0.802625411 | 1.184739646 |
| SPOCK2 | 0.979097431 | 0.800945764 | 0.830822195 | 1.153835063 |
| SH2D2A | 0.979297118 | 0.801898446 | 0.831645448 | 1.153163102 |
| GPSM3 | 1.021860508 | 0.807925662 | 0.858368514 | 1.216492546 |
| ADAM8 | 1.024478594 | 0.808750077 | 0.842273974 | 1.246098564 |
| FOLR2 | 0.981532575 | 0.808760183 | 0.84401042 | 1.141462442 |
| HLA.DOB | 0.982180788 | 0.812477683 | 0.846600079 | 1.13947438 |
| PSMB8 | 0.97804083 | 0.813695759 | 0.813124771 | 1.176404777 |
| DUSP2 | 0.98332564 | 0.819477095 | 0.851101425 | 1.136091759 |
| DOCK11 | 0.977779015 | 0.819725393 | 0.805951939 | 1.186239225 |
| CARD16 | 1.022840371 | 0.821279972 | 0.840837495 | 1.244238549 |
| LSP1 | 1.016934127 | 0.822458275 | 0.878195274 | 1.17759119 |
| GZMM | 0.9813018 | 0.824201039 | 0.830764003 | 1.159117655 |
| LGALS2 | 1.012384006 | 0.828498043 | 0.905698009 | 1.131636998 |
| CCL4L1 | 1.016376445 | 0.829454569 | 0.876726064 | 1.178271208 |
| DEF6 | 1.018278409 | 0.833350562 | 0.860178673 | 1.205436672 |
| LST1 | 0.982021933 | 0.835863204 | 0.827158728 | 1.165879104 |
| CORO1A | 0.985511848 | 0.84211686 | 0.853680659 | 1.137701307 |
| SRGN | 0.984671743 | 0.844522732 | 0.843811954 | 1.149045634 |
| HCST | 1.015051773 | 0.848189748 | 0.8710801 | 1.182819012 |
| CTLA4 | 1.017334921 | 0.848590683 | 0.852783117 | 1.213638404 |
| EVI2A | 1.0174417 | 0.852814069 | 0.847573599 | 1.221354244 |
| LAG3 | 0.986922146 | 0.861928027 | 0.850853956 | 1.144750302 |
| IRF8 | 1.014482817 | 0.872348228 | 0.851276486 | 1.20897899 |
| STAT1 | 1.012572675 | 0.873380826 | 0.868344059 | 1.180757112 |
| LCP1 | 1.012286092 | 0.874801487 | 0.869629873 | 1.178343986 |
| HLA.DQA1 | 0.990081808 | 0.877650849 | 0.872083555 | 1.124045948 |
| LIMD2 | 1.011531224 | 0.878223106 | 0.873546273 | 1.171312212 |
| PDCD1 | 0.989155985 | 0.880127107 | 0.858467687 | 1.139739535 |
| HCLS1 | 0.986443868 | 0.882300641 | 0.823381632 | 1.181798897 |
| ACP5 | 0.992290387 | 0.886515267 | 0.892232046 | 1.103569655 |
| APOBEC3D | 1.012453724 | 0.892046291 | 0.846736507 | 1.21060393 |
| JAK3 | 0.988708806 | 0.892172029 | 0.839004067 | 1.165125583 |
| VAV1 | 1.013126373 | 0.893543856 | 0.836976998 | 1.226347976 |
| SAMHD1 | 0.98513168 | 0.89355552 | 0.791043328 | 1.226841049 |
| FAM26F | 0.99140427 | 0.900496739 | 0.865924634 | 1.135066941 |
| ARHGDIB | 1.012704957 | 0.900624334 | 0.830662179 | 1.234643103 |
| HLA.DOA | 0.991492162 | 0.90463607 | 0.862153119 | 1.140234473 |
| RARRES1 | 1.006581264 | 0.907297188 | 0.90136244 | 1.124082606 |
| EVI2B | 0.991748786 | 0.919733099 | 0.844140428 | 1.165168284 |
| DOCK8 | 1.009287619 | 0.921765954 | 0.839245031 | 1.213783175 |
| CXCL11 | 0.993675011 | 0.924267846 | 0.871821868 | 1.132559373 |
| HLA.DMB | 1.006741788 | 0.930454637 | 0.865732709 | 1.170718186 |
| AOAH | 1.00685754 | 0.939602241 | 0.84370969 | 1.201553234 |
| MYO1F | 1.008353892 | 0.941241035 | 0.808245508 | 1.258005844 |
| ISG20 | 0.993172089 | 0.942328592 | 0.824918367 | 1.195743529 |
| RASSF2 | 1.006047652 | 0.943598966 | 0.851284624 | 1.188946504 |
| CLEC2B | 0.993134236 | 0.945451484 | 0.815262175 | 1.209814022 |
| ANXA1 | 0.99459661 | 0.946118062 | 0.849975991 | 1.163823952 |
| RAC2 | 1.004604668 | 0.946854637 | 0.877665979 | 1.149902769 |
| CD53 | 1.004787082 | 0.947946157 | 0.870580527 | 1.159682589 |
| SOCS1 | 1.004416061 | 0.948515441 | 0.878673703 | 1.148152744 |
| EPSTI1 | 1.004557354 | 0.951360286 | 0.868008394 | 1.162587233 |
| ETV7 | 0.997213191 | 0.969322879 | 0.865007664 | 1.149624667 |
| CMKLR1 | 1.00401014 | 0.970359226 | 0.812939805 | 1.239988933 |
| CCL4 | 0.997206609 | 0.972061418 | 0.852706004 | 1.166194463 |
| OAS2 | 1.001861519 | 0.980175582 | 0.865167417 | 1.160152917 |
| HSH2D | 1.000071845 | 0.999185824 | 0.871168679 | 1.148048271 |

# Supplementary Table 3 The GSEA results regarding the TIPRGPI risk groups (High vs Low)

| **NAME** | **SIZE** | **ES** | **NES** | **NOM p-val** | **FDR q-val** | **FWER p-val** |
| --- | --- | --- | --- | --- | --- | --- |
| GLYCOLYSIS | 197 | 0.460 | 1.841 | 0.000 | 0.186 | 0.085 |
| MTORC1_SIGNALING | 194 | 0.504 | 1.812 | 0.000 | 0.129 | 0.114 |
| MYC_TARGETS_V1 | 194 | 0.591 | 1.739 | 0.017 | 0.161 | 0.19 |
| UNFOLDED_PROTEIN_RESPONSE | 106 | 0.505 | 1.721 | 0.004 | 0.137 | 0.208 |
| G2M_CHECKPOINT | 189 | 0.604 | 1.700 | 0.028 | 0.124 | 0.226 |
| PROTEIN_SECRETION | 95 | 0.516 | 1.649 | 0.022 | 0.156 | 0.3 |
| MITOTIC_SPINDLE | 198 | 0.517 | 1.625 | 0.012 | 0.158 | 0.343 |
| PI3K_AKT_MTOR_SIGNALING | 103 | 0.465 | 1.619 | 0.006 | 0.141 | 0.35 |
| WNT_BETA_CATENIN_SIGNALING | 42 | 0.485 | 1.579 | 0.022 | 0.162 | 0.407 |
| E2F_TARGETS | 195 | 0.566 | 1.570 | 0.061 | 0.153 | 0.42 |
| MYC_TARGETS_V2 | 58 | 0.550 | 1.564 | 0.067 | 0.144 | 0.429 |
| DNA_REPAIR | 147 | 0.452 | 1.467 | 0.074 | 0.233 | 0.562 |
| P53_PATHWAY | 193 | 0.358 | 1.437 | 0.053 | 0.252 | 0.6 |
| UV_RESPONSE_UP | 154 | 0.345 | 1.428 | 0.054 | 0.244 | 0.615 |
| NOTCH_SIGNALING | 32 | 0.453 | 1.428 | 0.072 | 0.228 | 0.615 |
| REACTIVE_OXYGEN_SPECIES_PATHWAY | 47 | 0.421 | 1.406 | 0.105 | 0.240 | 0.636 |
| APOPTOSIS | 159 | 0.314 | 1.258 | 0.175 | 0.434 | 0.77 |
| ESTROGEN_RESPONSE_EARLY | 194 | 0.294 | 1.252 | 0.156 | 0.421 | 0.778 |
| ANDROGEN_RESPONSE | 96 | 0.333 | 1.240 | 0.209 | 0.415 | 0.786 |
| SPERMATOGENESIS | 132 | 0.316 | 1.238 | 0.167 | 0.398 | 0.79 |
| APICAL_JUNCTION | 193 | 0.305 | 1.223 | 0.193 | 0.402 | 0.801 |
| TGF_BETA_SIGNALING | 54 | 0.382 | 1.223 | 0.259 | 0.384 | 0.801 |
| HYPOXIA | 190 | 0.296 | 1.184 | 0.239 | 0.427 | 0.822 |
| HEME_METABOLISM | 191 | 0.293 | 1.153 | 0.302 | 0.452 | 0.831 |
| HEDGEHOG_SIGNALING | 35 | 0.364 | 1.103 | 0.353 | 0.505 | 0.861 |
| IL2_STAT5_SIGNALING | 194 | 0.275 | 1.093 | 0.338 | 0.503 | 0.863 |
| ANGIOGENESIS | 36 | 0.333 | 1.062 | 0.377 | 0.526 | 0.871 |
| TNFA_SIGNALING_VIA_NFKB | 198 | 0.304 | 1.053 | 0.397 | 0.522 | 0.875 |
| UV_RESPONSE_DN | 138 | 0.275 | 0.995 | 0.460 | 0.593 | 0.9 |
| ESTROGEN_RESPONSE_LATE | 196 | 0.218 | 0.975 | 0.498 | 0.605 | 0.904 |
| APICAL_SURFACE | 43 | 0.288 | 0.972 | 0.476 | 0.591 | 0.904 |
| COMPLEMENT | 200 | 0.243 | 0.946 | 0.508 | 0.609 | 0.913 |
| INFLAMMATORY_RESPONSE | 197 | 0.269 | 0.910 | 0.541 | 0.648 | 0.927 |
| IL6_JAK_STAT3_SIGNALING | 87 | 0.274 | 0.907 | 0.569 | 0.633 | 0.927 |
| PEROXISOME | 104 | 0.244 | 0.903 | 0.567 | 0.623 | 0.929 |
| KRAS_SIGNALING_UP | 194 | 0.220 | 0.889 | 0.608 | 0.624 | 0.934 |
| EPITHELIAL_MESENCHYMAL_TRANSITION | 197 | 0.260 | 0.877 | 0.588 | 0.624 | 0.938 |
| ADIPOGENESIS | 193 | 0.241 | 0.876 | 0.576 | 0.608 | 0.938 |
| MYOGENESIS | 197 | 0.207 | 0.848 | 0.705 | 0.633 | 0.946 |
| CHOLESTEROL_HOMEOSTASIS | 73 | 0.235 | 0.840 | 0.600 | 0.627 | 0.947 |

# Supplementary Table 4 Molecular docking scores of the top six compounds.

| **Rank** | **ZINC ID** | **Compound name** | **Glide gscore** | **2D-structure** |
| --- | --- | --- | --- | --- |
| 1 | ZINC000003806262 | Pentostatin | -7.787 | 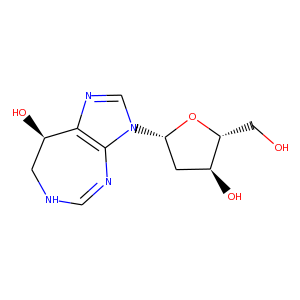 |
| 2 | ZINC000001843030 | Allantoin | -6.834 | 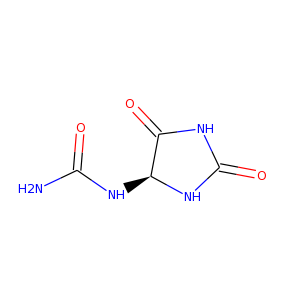 |
| 3 | ZINC000003812887 | Mizoribine | -6.525 | 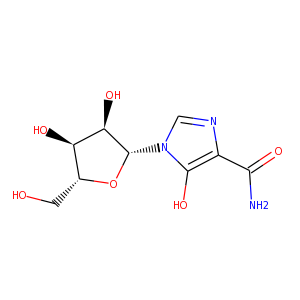 |
| 4 | ZINC000001529214 | Xylose | -6.437 | 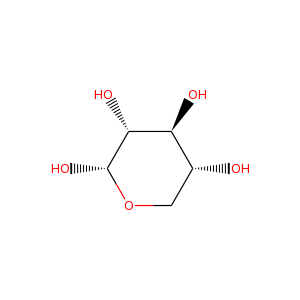 |
| 5 | ZINC000003995890 | Deoxynojirimycin | -6.302 | 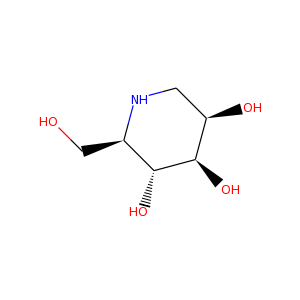 |
| 6 | ZINC000021981208 | 6-Hydroxyetodolac | -6.292 | 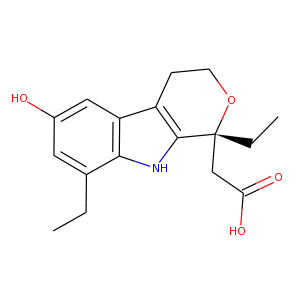 |

**Supplementary Figures**


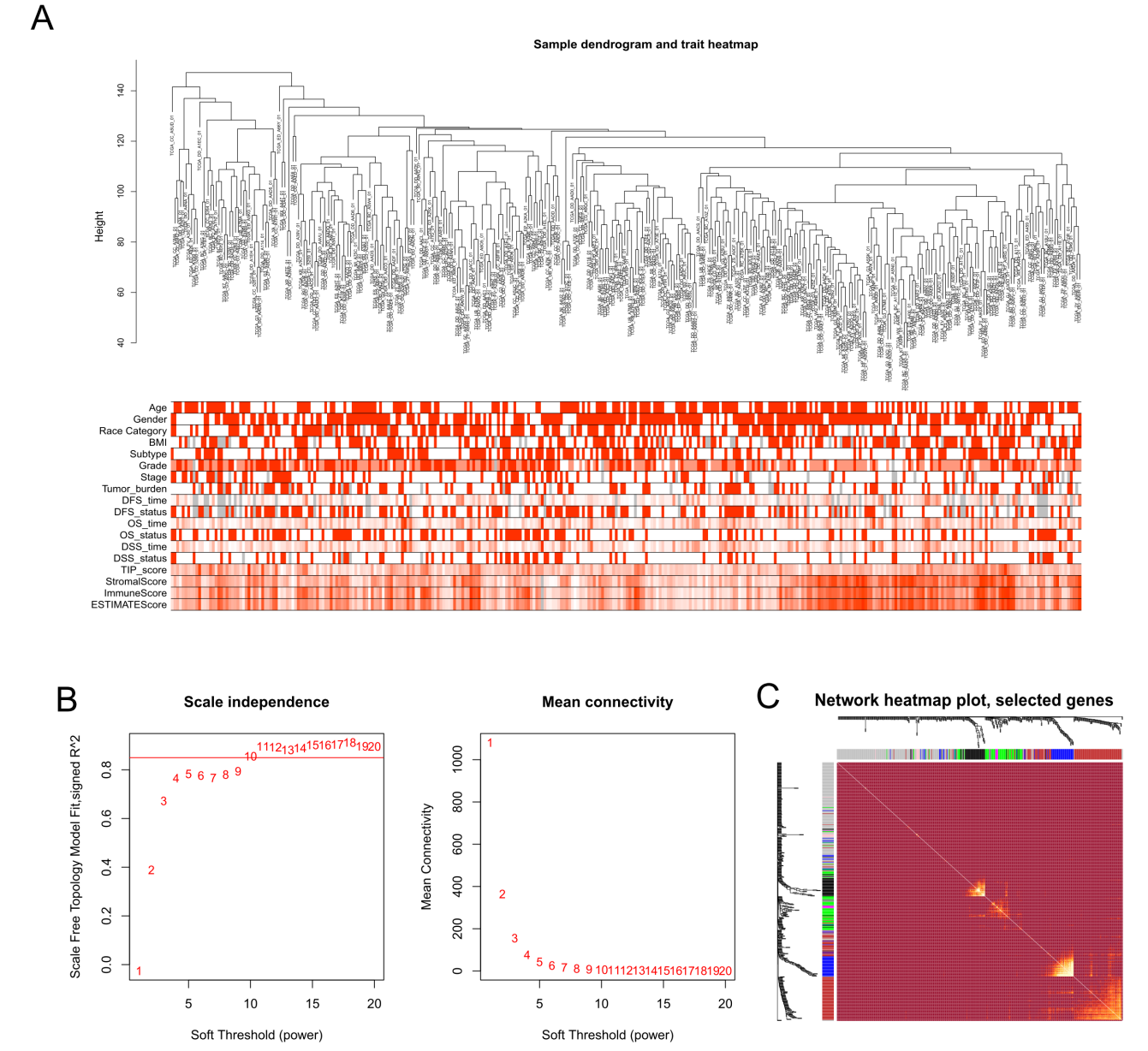


**Supplementary Figure 1 Building a scale-free network with the optimal soft-thresholding power using the MAD top 5000 genes of the TCGA-LIHC dataset.** (A) Sample clustering tree with clinicopathological features. (B) Choosing the optimal soft-thresholding power from a wide range. (C) The constructed eigengene network displaying the clustering tree with the dissimilarity and intercorrelation among each module.

.


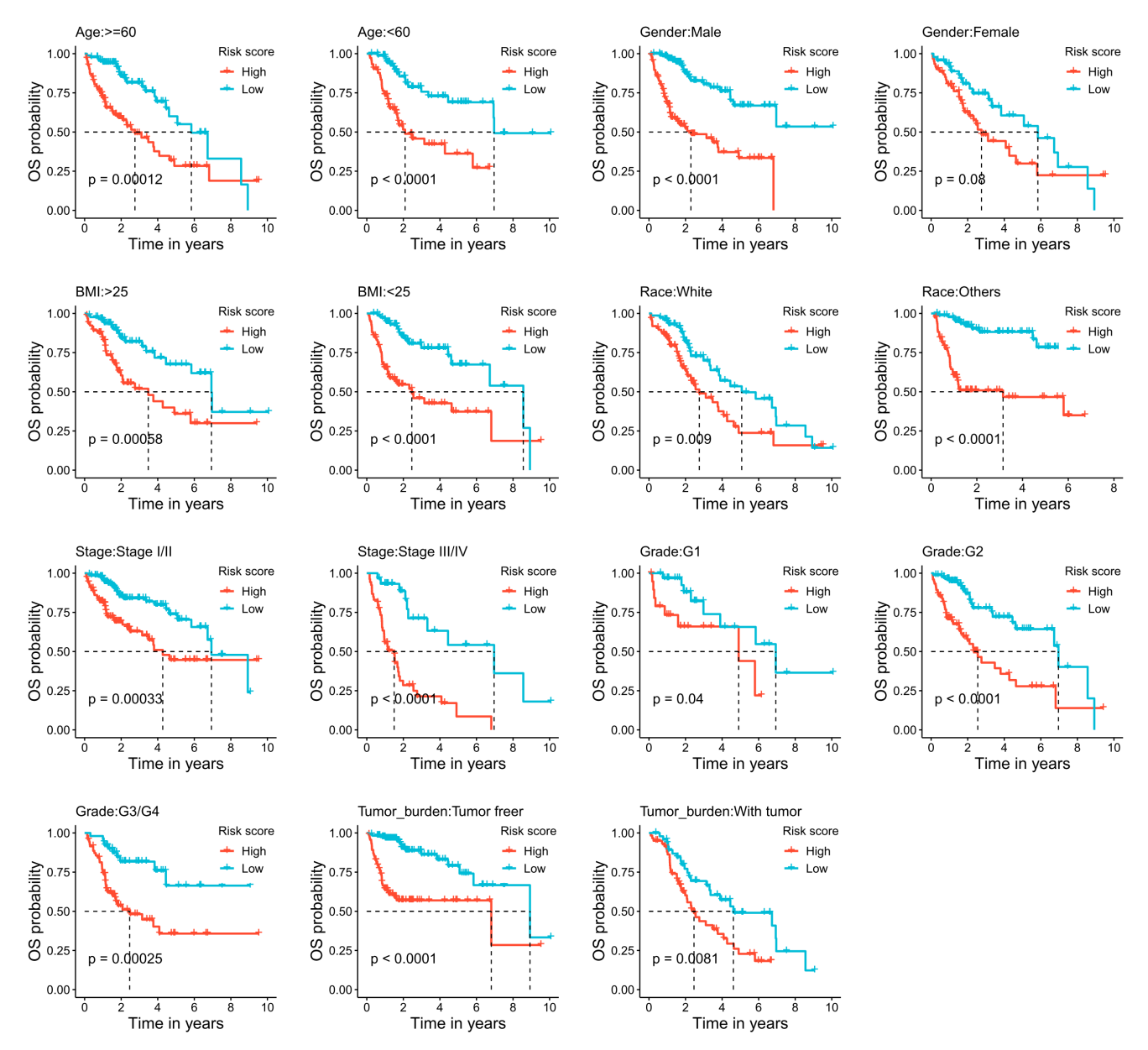


**Supplementary Figure 2 Kaplan-Meier curve illustrates the prognostic value of TIPRGPI risk score signature based on the subgroups of different clinicopathological variables.**


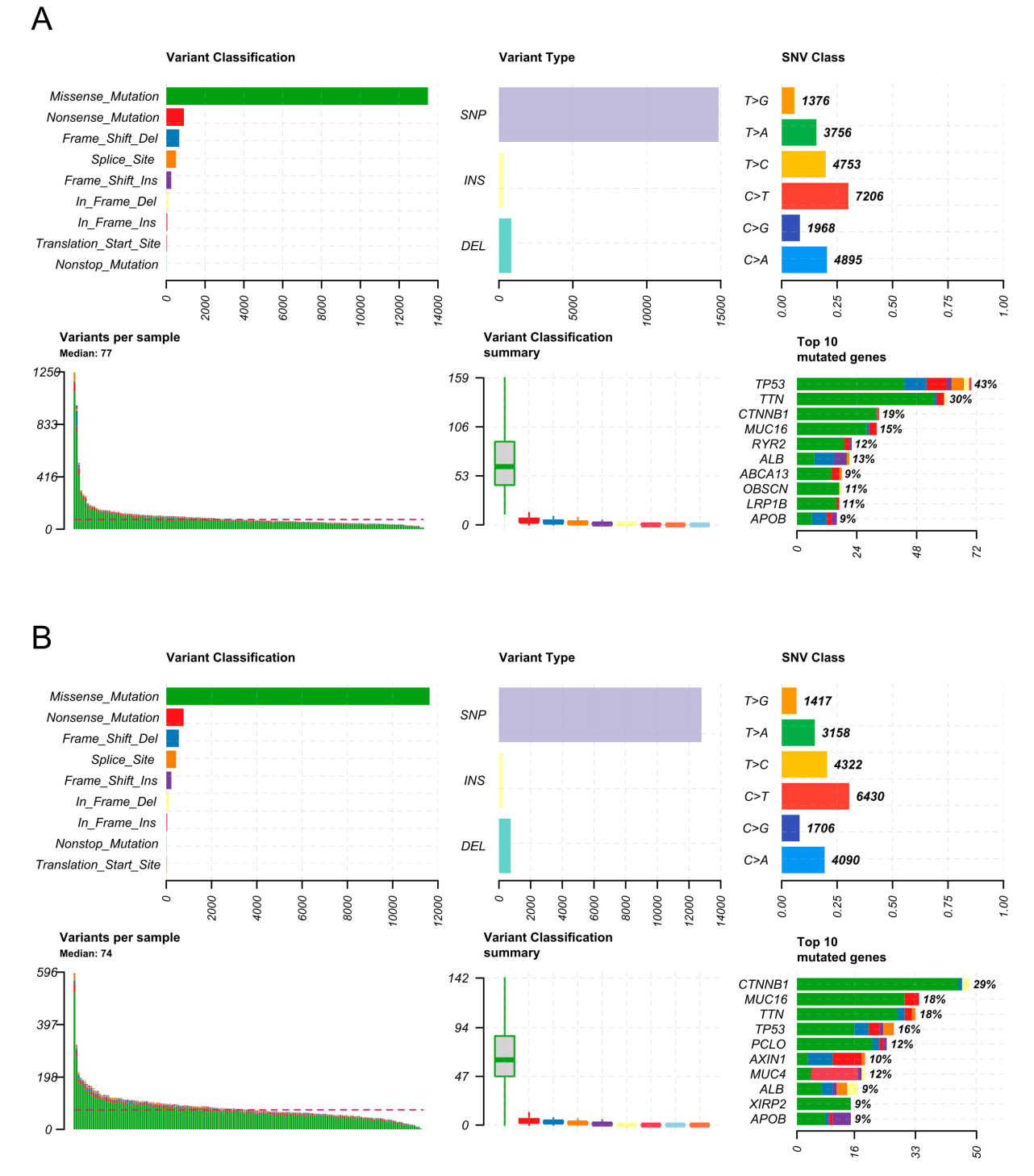


**Supplementary Figure 3 Summary of the mutation information in the high- (upper) and low- (bottom) risk groups with statistical calculations.**


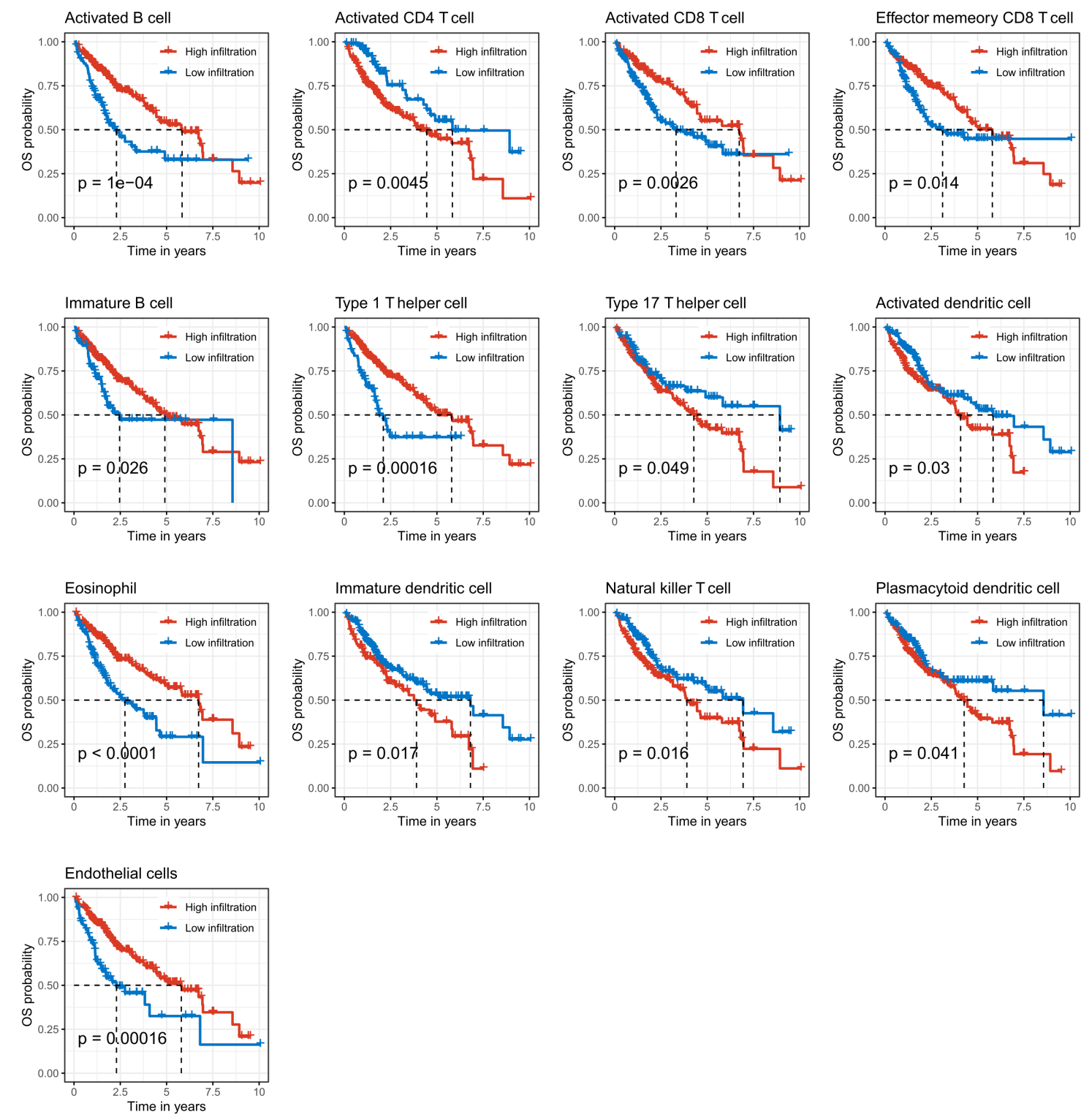


**Supplementary Figure 4 Significant associations of the 13 TME cells infiltration and OS outcomes for HCC patients identified by Kaplan-Meier analysis.**


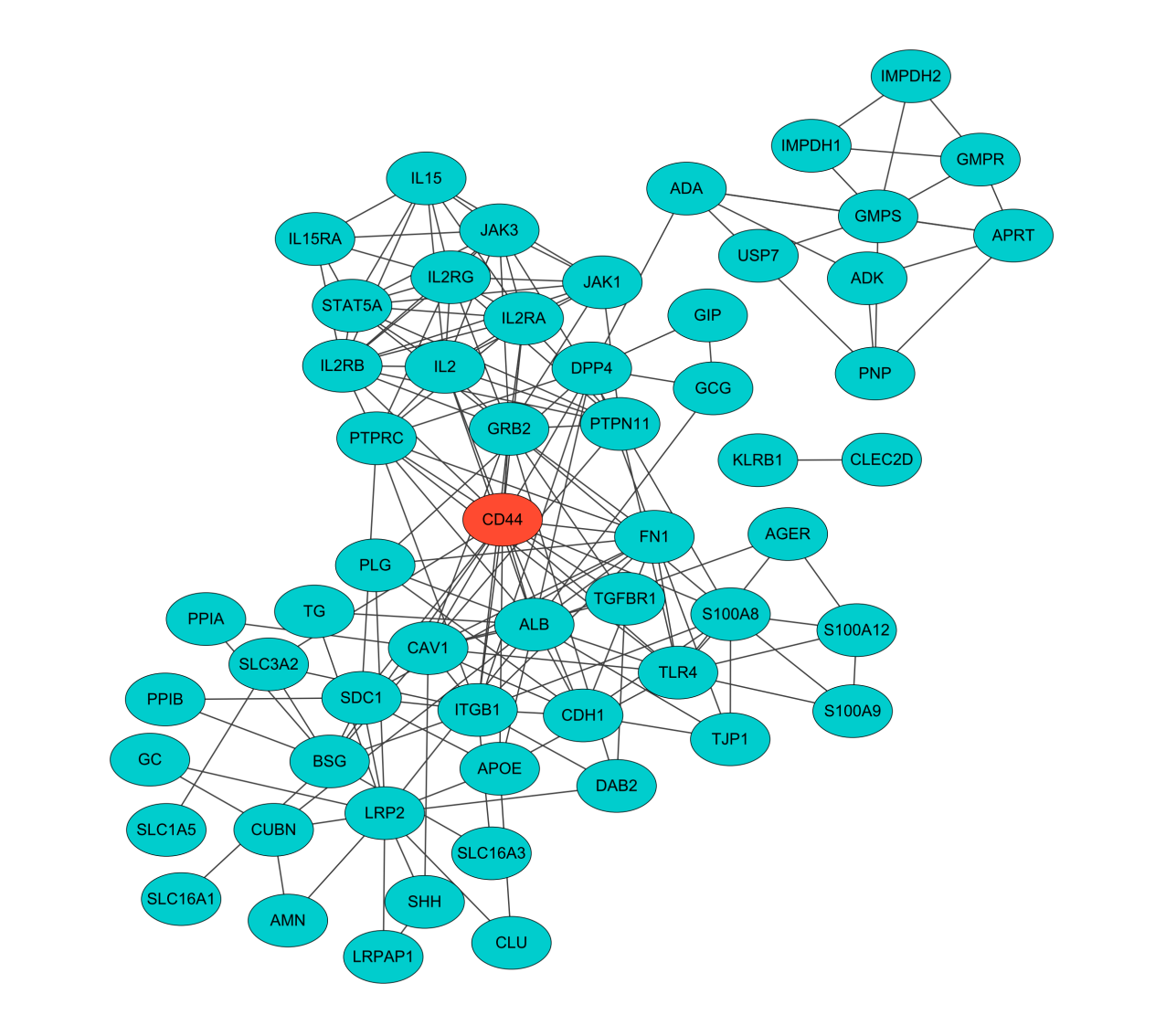


**Supplementary Figure 5 PPI network of the TIPRGPI identifying CD44 as the hub gene with the highest degree. The red node indicates the selected hub gene, and the green nodes indicate**

**the other genes consisting the network.**
